# Deep brain stimulation of the basolateral amygdala for treatment-refractory combat post-traumatic stress disorder (PTSD): study protocol for a pilot randomized controlled trial with blinded, staggered onset of stimulation

**DOI:** 10.1186/1745-6215-15-356

**Published:** 2014-09-10

**Authors:** Ralph J Koek, Jean-Philippe Langevin, Scott E Krahl, Hovsep J Kosoyan, Holly N Schwartz, James WY Chen, Rebecca Melrose, Mark J Mandelkern, David Sultzer

**Affiliations:** Psychiatry Service, VA Greater Los Angeles Healthcare System (VAGLAHS), 11301 Wilshire Blvd, Los Angeles, CA 90073 USA; David Geffen School of Medicine at UCLA, Los Angeles, USA; Neurosurgery Service, VAGLAHS, 11301 Wilshire Blvd, Los Angeles, C 90073 USA; Research and Development Service, VAGLAHS, 11301 Wilshire Blvd, Los Angeles, CA 90073 USA; Neurology Service, VAGLAHS, 11301 Wilshire Blvd, Los Angeles, CA 90073 USA; Brain, Behavior, and Aging Research Center, VAGLAHS, 11301 Wilshire Blvd, Los Angeles, CA 90073 USA; Imaging Department, Radiology Service, VAGLAHS, 11301 Wilshire Blvd, Los Angeles, CA 90073 USA; Physics Department, UC Irvine, Irvine, CA 92697 USA; 16111 Plummer St. (116A-11), North Hills, CA 91343 USA

**Keywords:** Stress disorders, Post-traumatic, Veteran’s health, Deep brain stimulation, Amygdala, Positron emission tomography, Electroencephalograms, Caregivers, Controlled clinical trial, Neuropsychiatry

## Abstract

**Background:**

Combat post-traumatic stress disorder (PTSD) involves significant suffering, impairments in social and occupational functioning, substance use and medical comorbidity, and increased mortality from suicide and other causes. Many veterans continue to suffer despite current treatments. Deep brain stimulation (DBS) has shown promise in refractory movement disorders, depression and obsessive-compulsive disorder, with deep brain targets chosen by integration of clinical and neuroimaging literature. The basolateral amygdala (BLn) is an optimal target for high-frequency DBS in PTSD based on neurocircuitry findings from a variety of perspectives. DBS of the BLn was validated in a rat model of PTSD by our group, and limited data from humans support the potential safety and effectiveness of BLn DBS.

**Methods/Design:**

We describe the protocol design for a first-ever Phase I pilot study of bilateral BLn high-frequency DBS for six severely ill, functionally impaired combat veterans with PTSD refractory to conventional treatments. After implantation, patients are monitored for a month with stimulators off. An electroencephalographic (EEG) telemetry session will test safety of stimulation before randomization to staggered-onset, double-blind sham versus active stimulation for two months. Thereafter, patients will undergo an open-label stimulation for a total of 24 months. Primary efficacy outcome is a 30% decrease in the Clinician Administered PTSD Scale (CAPS) total score. Safety outcomes include extensive assessments of psychiatric and neurologic symptoms, psychosocial function, amygdala-specific and general neuropsychological functions, and EEG changes. The protocol requires the veteran to have a cohabiting significant other who is willing to assist in monitoring safety and effect on social functioning. At baseline and after approximately one year of stimulation, trauma script-provoked ^18^FDG PET metabolic changes in limbic circuitry will also be evaluated.

**Discussion:**

While the rationale for studying DBS for PTSD is ethically and scientifically justified, the importance of the amygdaloid complex and its connections for a myriad of emotional, perceptual, behavioral, and vegetative functions requires a complex trial design in terms of outcome measures. Knowledge generated from this pilot trial can be used to design future studies to determine the potential of DBS to benefit both veterans and nonveterans suffering from treatment-refractory PTSD.

**Trial registration:**

PCC121657, 19 March 2014.

**Electronic supplementary material:**

The online version of this article (doi:10.1186/1745-6215-15-356) contains supplementary material, which is available to authorized users.

## Background

### Overview

The present paper discusses the rationale and methodology for experimental use of deep brain stimulation (DBS) for treatment-refractory combat post-traumatic stress disorder (PTSD). DBS is an invasive treatment, and the potential for benefits must clearly outweigh the risks. Potential benefits exist for patients whose illness does not respond to currently available, lower risk treatments and continue to suffer severe symptomatic and functional impairment, and these benefits depend on the plausibility of benefit from direct neurocircuitry modulation with DBS electrodes. Risks include those of the surgical procedure, implanted hardware, and of stimulation, along with potential ethical and social implications. This paper outlines our weighing of these considerations in the preparation of a first-ever trial of DBS for PTSD, targeting the basolateral nucleus of the amygdala (BLn).

### The risks of chronic, treatment-resistant combat post-traumatic stress disorder

PTSD is a serious psychiatric condition that affects an estimated 6.8% of the US population at some point in their lifetime[1] with a 12-month prevalence of 3.5%[2]. Military combat is the classic precipitant of PTSD, and the prevalence of the condition is significantly greater among combat veterans than in the general population. Lifetime/current prevalence in Vietnam veterans in a well-known 1990 survey was 30.9%/15.2% for men and 26.9%/8.1% for women[3]. In Gulf War veterans, 12.1% of a sample of 30,000 had a current diagnosis of PTSD[4]. Among Operation Iraqi Freedom/Operation Enduring Freedom (OIF/OEF) veterans, one investigation[5] found that 13% met criteria for PTSD at some point after discharge, while in two other studies, 13.8% and 14% met criteria for current PTSD[6, 7]. A 2008 study found that PTSD or depression among troops deployed to Iraq and Afghanistan cost the US government $6.2 billion[6]. Persistent severe PTSD was found to be associated with significant distress and dysfunction.

PTSD is defined[8, 9] by the occurrence of distressing or disabling perceptual, emotional, and behavioral changes that persist after an experience in which the sufferer has witnessed or been threatened with death or severe injury. In the 3rd and 4th editions of the American Psychiatric Association’s (APA) Diagnostic and Statistical Manual of Mental Disorders (DSM-III, IIIR, IV and IV-R), PTSD was characterized by three clusters of psychiatric symptoms. The first, re-experiencing, involves the emotional and perceptual reliving of traumatic event(s) either spontaneously or in response to ‘triggers’ that remind one of the event because they bear some similarity to the original circumstance. The next symptom cluster, avoidance and numbing, involves the tendency to social isolation and reduced ability to experience positive emotions in relationships with others. The hyperarousal cluster includes hypervigilance about one’s surroundings, sleep disturbance, anxiety, and anger dyscontrol, including physical violence. In the recently published DSM-5, a 4th cluster of symptoms, called ‘negative alterations in cognitions and mood’, has been added. This incorporates several symptoms previously included in the DSM-IV avoidance and numbing cluster, and adds persistent distorted blame of self or others, and persistent negative emotional state as new symptoms, based on empirical data on the phenomenology of the condition published since DSM-IV[10]. Another new symptom, reckless or self-destructive behavior, was added to the hyperarousal cluster, now termed ‘alterations in arousal and reactivity’.

Individuals with this condition, particularly PTSD due to combat, suffer terribly[11]. Among Vietnam veterans, chronic PTSD is associated with less life satisfaction and happiness[12], increased rates of major depression[13], impaired family functioning[12], marital problems[14], occupational disability[11], substance use disorders[15], general medical illnesses[16], and suicide[17] compared with the general population. One sobering study found a 17% mortality rate over six years of follow-up in 51 Vietnam combat veterans, despite intensive treatment at the National Center for PTSD in New Haven (NCPTSD)[18]. PTSD with psychotic features may characterize the most severely ill individuals with this condition[19]. Unfortunately, PTSD in OIF/OEF veterans is associated with social and medical morbidity similar to that suffered by Vietnam veterans[20–23].

Current treatments for PTSD include psychotropic medications and/or psychotherapy. Antidepressants are commonly prescribed. The selective serotonin reuptake inhibitor (SSRI) antidepressants, sertraline and paroxetine, are the only US Food and Drug Administration (FDA) approved medications for the condition. It is worth noting, however, that <10% of patients in the trials leading to FDA approval were combat PTSD sufferers[24–27], and at least three trials in combat PTSD failed to demonstrate better efficacy with an SSRI than placebo[28, 29]. Recent guidelines conclude that the evidence now does not provide strong support for the use of SSRIs for combat PTSD[30, 31]. Venlafaxine, in a mixed-trauma population[32], and prazosin, in veterans and active duty military personnel[33, 34], have both shown efficacy in placebo-controlled trials, and several other medications have at least open-label findings in favor of benefit for combat veterans. But, except for the most recent study of prazosin[34], none of these have demonstrated efficacy in large randomized controlled trials (RCTs). Some commonly used pharmacologic strategies, including second-generation antipsychotic augmentation of unsuccessful antidepressant therapy, as well as divalproex and bupropion, have failed to separate from placebo in RCTs with combat vets, and one very commonly used medication class - benzodiazepines - while widely used in clinical settings, has no good supporting evidence, and is described as not effective and potentially harmful in the recent NCPTSD treatment guideline[31].

Individual or group psychotherapies are also provided for veterans with PTSD. The NCPTSD considers Prolonged Exposure therapy (PE)[35–37], Cognitive Processing Therapy (CPT)[38, 39], and Eye Movement Desensitization and Reprocessing (EMDR)[40–42] treatments of proven efficacy. Each of these evidence-based therapies involves a component of exposing patients to anxiety-evoking reminders of the traumatic experience(s). Exposure-based treatments are found by most investigators to be the most effective therapeutic interventions[43–45]. This was also the position of the Institute of Medicine’s 2007 report[46]. Recently, Eftekhari and colleagues[47] published results from a national roll-out of Prolonged Exposure Therapy (PE) for combat-related PTSD carried out in a manualized, but open-label fashion by United States Department of Veterans Affairs practitioners across the country. Nearly 50% of patients achieved the response criterion of 50% reduction in PTSD according to the PTSD Checklist-Military Version[48] after a mean of 11.6 weekly sessions. In a separate report, Goodson and colleagues[49] also found significant benefit with PE among a group of veterans with predominantly combat PTSD. Tuerk *et al*. (2011)[50] also found PE effective for PTSD in OEF/OIF combat veterans.

Combat PTSD is generally more severe and has a lower rate of remission than noncombat PTSD[51, 52]. A meta-analysis of controlled psychotherapy and psychopharmacology trials found that overall, nearly 2/3 of patients treated with exposure therapy who completed treatment no longer met criteria for PTSD, but treatment response was significantly poorer in combat compared to noncombat PTSD[53]. In a study of Hispanic and American Indian veterans, only 20% of 106 with combat PTSD had experienced a year or longer without symptoms since onset, in contrast to 40% of veterans with PTSD from noncombat trauma[51]. In a long-term follow-up study of over 200 Israeli combat veterans, 26 to 50% were still diagnosed with PTSD 20 years later[54]. In OEF/OIF veterans, PTSD is an important predictor of impaired psychological and physical functioning after deployment[55]. Neuropsychological deficits in OIF veterans worsened in proportion to severity of PTSD when assessed one year after return from deployment[56]; and in another study, PTSD mediated the effect of traumatic brain injury (TBI) on psychosocial function in OEF/OIF veterans[57]. In OEF/OIF veterans with both mild TBI (mTBI) and PTSD, PTSD accounts for more impairment on neuropsychological testing than mTBI[58, 59]. Overall, long-term outcomes with even the most intensive treatments reveal persistent suffering and disability for many veterans, even when there is significant symptomatic improvement[60–62].

New treatments are sorely needed for the large number of patients who are left with significant disability and suffering despite the best current care. Intrusive and hyperarousal cluster symptoms are in particular more severe in combat PTSD[63, 64], and decreased re-experiencing symptoms over time is associated with improvement in occupational, social, and family functioning[65]. Our novel strategy, involving direct neuromodulation, targets the amygdala based on research that demonstrates an association of intrusive and cue-induced hyperarousal symptoms with overactivity in this brain region. If the results of this project are positive, it will have direct benefit for veterans suffering from treatment-resistant PTSD and will contribute to understanding the pathophysiology of the condition.

### Deep brain stimulation

Deep brain stimulation (DBS) refers to the process of delivering an electrical current to a precise location in the brain. DBS is now a common clinical practice and more than 100,000 patients have been implanted[66]. The FDA has granted approval for DBS for Parkinson’s disease and essential tremor, the first conditions to have shown benefit in systematic investigations comparing DBS to best medical therapy. Surgical targeting is based on specific neurocircuitry models. Because of the significant long-term benefit, including reduced mortality compared to alternatives in treatment-refractory Parkinson’s Disease[67], DBS has largely replaced targeted ablative neurosurgery.

For psychiatric disorders, DBS was also first studied in conditions for which targeted ablative neurosurgery has been used to treat the most severely ill patients, who do not respond to available, less invasive treatments. With the first reported cases in obsessive-compulsive disorder (OCD) in 2003[68] and major depression in 2005[69], DBS was found to have the capacity to produce response in patients who had previously been refractory to all standard interventions. In OCD, long-term benefit has been repeatedly demonstrated across different investigative centers[70–72], such that the US FDA granted a Humanitarian Device Exemption for Anterior Limb of the Internal Capsule (ALIC) DBS on a case-by-case basis. Beneficial effects for treatment-refractory depression have also been replicated by investigators in various countries[73–77], but DBS for this condition remains experimental. Reviewers have found response and remission rates in both conditions in the range of 25 to 50%[78–80] with benefits maintained or accrued over at least the first three years, and significant improvement in functioning as well as symptomatology[70, 81, 82]. Other psychiatric conditions in which DBS is under active study include addiction[83], Alzheimer’s disease[84], and anorexia nervosa[85].

In its current application, DBS electrical current is modulated by frequency, pulse width, and amplitude. These parameters can be modified postoperatively with an external programmer. Reversibility is an obvious benefit of this treatment over resection or a destruction procedure. While the exact mechanism of action is not yet fully understood, it is relevant that the brain is a voltage sensitive organ, composed of numerous ion channels with different voltage sensitivity and activation ranges. The neurophysiological responses from an electrical stimulation can be determined from the voltage changes at a specific tissue site and the subsequent activations of ion channels. Variations of how the stimulating current is delivered, such as by changing the stimulation pulse width, the amplitude and the frequency, would result in differences in the extent of tissues that could reach the target voltage. The stimulated tissues could be modeled as a sphere with the tip of the stimulation electrode at the center. The stimulation effect, or the induced voltage change, is impedance dependent and tapers down, moving further away from the center of stimulation. After a tissue is stimulated, the activation of various ion channels subsequently plays an active role in determining the final neurophysiological effects[86]. Clinically, we can approximate the stimulated effect as pulse width determines the surface area of neural tissue covered by the stimulation, amplitude determines the strength of the effect, and frequency determines activation or inhibition. High frequency stimulation blocks the activity of the neural structure and clinically mimics the effects of a lesion[87–89], although electrophysiological monitoring has shown that neural fibers can be activated even with high-frequency DBS[87]. A current model holds that DBS adds a significant amount of interference to the final output signal from the nucleus, therefore rendering the output signal meaningless. This process is referred to as ‘frequency jamming’. Several other hypotheses of DBS mechanisms have been put forward, and it is likely that mechanisms of action differ in different conditions and targets. In each of the psychiatric conditions in which it has been studied, DBS targets have been chosen based on functional neuroimaging findings of abnormal brain circuitry. In both depression[73] and OCD[90], high-frequency DBS targeted to an area of hyperactivity normalizes metabolism in interconnected brain regions. Similarly, in Alzheimer’s Disease, functional connectivity with hippocampus and cerebral cortex increased after 1 year of DBS in the nucleus basalis[91].

### Rationale for targeting the amygdala with deep brain stimulation in post-traumatic stress syndrome

Our planned intervention is based on a well-accepted model of the pathophysiology of PTSD involving amygdala hyperactivity in association with reminders of the traumatic event[92]. Research in animal models has shown that the amygdala mediates emotional and autonomic responses to environmental stimuli generally, and conditioned fear responses specifically. The amygdala is a complex, heterogenous gray matter structure in the medial temporal lobes. It is composed of several subdivisions with different functional roles. Two principal subdivisions - the lateral nucleus and the central nucleus - can be conceived of as the receptive and expressive regions of the amygdaloid complex. The basolateral nucleus (BLn) acts as a relay nucleus within the amygdala by receiving multiple connections from the lateral nucleus and sending efferents to the central nucleus[93]. The BLn forms a connectivity loop with the medial prefrontal cortex (mPFC)[94]. This reciprocal connection is thought to be involved in the cortical (that is, top-down) control of emotions. The function of the amygdala is to link sensory input to emotional responses that then guide behavior. The lateral nucleus screens sensory input, the BLn modulates (suppresses or enhances) these inputs, and the central nucleus orchestrates the emotional response to the inputs. Functional neuroimaging studies in humans also suggest that the amygdala mediates the acquisition, consolidation, and extinction of conditioned fear responses. When PTSD patients are subjected to imagery or sounds reminiscent of their trauma and scanned with functional magnetic resonance imaging (fMRI), positron emission tomography/computed tomography (PET-CT or PET), or single photon emission computerized tomography (SPECT), there is overactivity in the amygdala compared to normal controls[95–108]. A meta-analysis of these functional neuroimaging studies found that the focus of hyperactivity is located in the basal portion of the amygdala[109]. These authors and other reviewers[110] found that amygdala hyperactivity is associated with conditioned anxious hyperarousal in several anxiety disorders, in particular, anxiety disorders in which pathological anxiety responses are linked to specific environmental stimuli (PTSD, social anxiety disorder, and specific phobia). A more recent meta-analysis of functional neuroimaging studies involving symptom provocation in PTSD patients revealed hyperactivation of the bilateral amygdalae, as well as in midline retrosplenial cortex, precuneus, and pregenual/anterior cingulate gyrus in response to trauma-related stimuli[111]. Also very recently, Yan and colleagues[112] found increased amygdala activity in the resting state in combat veterans with PTSD, compared to those without PTSD. In PTSD, the intensity of BOLD signals on fMRI and regional blood flow on ^15^O_2_ PET in the amygdala are positively associated with symptom severity[102, 113].

Several authors[114–116] have found that successful cognitive-behavioral treatment of PTSD is associated with a reduction in pretreatment amygdala hyperactivity. Correspondingly, another study found that higher pretreatment amygdala activation predicted lower likelihood of response to exposure therapy in PTSD[113]. Thomaes *et al*.[117] recently reviewed four randomized controlled trials and five pre-post studies and found that in adult-onset trauma-related PTSD, there are decreased amygdala and increased dorsolateral prefrontal activations after treatment.

An elegant study using a different methodological approach has even suggested a causal relationship between amygdala activation and the development of PTSD in combat veterans. Koenigs and colleagues[118] separated veterans in the Vietnam Head Injury Study (VHIS) into four groups based on the location of their brain damage and compared the lifetime prevalence of PTSD in each group with that in a group with no brain injury. In combat veterans without brain injury, the prevalence of PTSD was 48%. In those with brain damage sparing both the vmPFC and amygdala, PTSD prevalence was similar at 40%. Most notably, none of the 15 veterans with amygdala damage ever developed PTSD. To further evaluate the specificity of amygdala damage, the authors looked at a subgroup with temporal lobe lesions sparing the amygdala. In this group, the rate of PTSD was similar to that in the non-brain-injured group at 32%[118]. Complementary results have been seen with structural MRI studies, in which combat veterans with, versus those without, PTSD have been shown to have larger amygdala volumes[119], and smaller volumes of the subgenual anterior cingulate (sgACC), caudate, hypothalamus, left insula, left middle temporal gyrus (MTG), and right middle frontal gyrus. In veterans with PTSD, CAPS scores correlate inversely with volumes of the sgACC, caudate, hypothalamus, insula, and left MTG[120].

Fear extinction is likely subserved by a ventromedial prefrontal cortex (vmPFC) projection to the amygdala. The vmPFC has a reciprocal connection with the amygdala, and within the amygdala, the BLn forms the main circuit loop with the vmPFC[121]. The vmPFC projection to the BLn has been shown in animals to provide an inhibitory effect on amygdala activation with extinction of conditioned fear responses, the latter being mediated by amygdala output nuclei that receive projections from the BLn. In addition, the BLn is the primary locus of memory for aversive conditioned stimulus-unconditioned stimulus (CS-US) associations[94]. Thus, the inhibitory role of the vmPFC on the amygdala is likely to be mediated through the BLn. In those terms, failure of fear extinction in PTSD can be understood as a failure of inhibition of the BLn by the vmPFC. Therefore, inhibiting BLn hyperactivity should restore normal fear extinction in PTSD patients. In a rat model, enhanced extinction of a conditioned fear response by the NMDA-receptor glutamatergic agonist, d-cycloserine, was localized to the BLn[122].

In humans, the functional neuroimaging review by Etkin and Wager[109] showed significant hypoactivation of the vmPFC in PTSD studies, but not in other conditions (for example, social anxiety disorder and specific phobia) that also have cue-related fear arousal in association with amygdala hyperactivation. Resting state fMRI studies of combat veterans with PTSD completed since we initially developed this protocol have confirmed and extended this pattern. In a study of OEF/OIF combat veterans using the amygdala as a seed, Sripada and colleagues[123] demonstrated increased amygdala connectivity with the insula and hypothalamus, and reduced functional connectivity with the dorsal and rostral anterior cingulate cortex, in those with, compared to those without, PTSD. Rabinak *et al*.[124] also showed increased amygdala-insula connectivity. Hayes and colleagues[125] found a direct correlation between amygdala hyperactivity and vmPFC hypoactivation in a quantitative meta-analysis of 26 fMRI and PET symptom-provocation studies in adult PTSD, even though their meta-analysis demonstrated amygdala hyperactivity in studies with cognitive-emotional, but not symptom -provocation tasks. Most recently, Brown *et al*.[126] found that combat-exposed veterans with, compared to those without, PTSD had reduced functional connectivity between the BLn specifically, and prefrontal regions mediating cognitive control of emotional responses.

Taken together, the findings reviewed suggest the possibility that patients whose PTSD symptoms do not respond to exposure therapy or other interventions may have unremitting amygdala hyperarousal that is resistant to, or insufficiently suppressed by, cognitive efforts normally mediated by vmPFC activation.

### Preclinical validation studies of amygdala deep brain stimulation in post-traumatic stress disorder

Based on the foregoing, it is predicted that high-frequency DBS of the BLn will reduce the symptoms of PTSD. We tested this hypothesis in a rat model using inescapable shocks. Inescapable shocks in rats produce long-lasting behavior changes that mimic PTSD[127]. Mikics and colleagues[128] demonstrated that rats traumatized by inescapable shocks, in the presence of a conspicuous object, had the tendency to bury the object when re-exposed to it 28 days later. Burying behavior does not occur normally in rats. The behavior models PTSD faithfully in that it is maintained over several weeks without signs of extinction, it is generalized to other objects than the one originally presented and the rats also displayed sustained social avoidance[128]. The behavior is robust and the majority of rats will have buried the object completely at the end of a 10-minute observation period, making this model attractive for studies.

We conducted two rodent studies using this PTSD model and high-frequency BLn DBS as a treatment. In the first study[129], we showed that DBS of the right BLn corrected the object-burying behavior following inescapable shocks in rats. The difference in behavior was striking. The sham control rats spent on average 13 times more time burying the ball than the DBS-treated rats (*P* <0.005), while the latter spent 18 times more time exploring the ball. In the second study, we showed that BLn DBS, but not paroxetine, reduced burying behavior following inescapable shocks[130]. In that study, we used a crossover design with real versus sham DBS, each administered for one week. We demonstrated that 1) BLn DBS was effective at one week, and the effects persisted after another week of sham treatment, and 2) BLn DBS was efficacious when the initiation of the therapy was delayed for a week after establishment of the behavior, modeling chronic PTSD. In addition we tested whether the effect of DBS on ball burying behavior was due to a general pattern of behavioral inhibition. This was assessed using the elevated plus maze, a model for assessing general anxiety, following the last burying behavior evaluation at 14 days. Rats treated with paroxetine spent significantly more time in the open arms, suggesting a reduction in general anxiety. The animals treated with DBS showed no significant difference. These results suggest that paroxetine carries its therapeutic effects on PTSD through a nonspecific reduction in general anxiety, while DBS improves PTSD without such nonspecific anxiety-reducing effects. The DBS-treated rats did not exhibit signs of fearlessness or carefree behavior during the trial. The animals treated with DBS in this study also did not display abnormal or violent behavior.

Other preclinical studies[131, 132] have reported that DBS of the amygdala and the hippocampus respectively raised the threshold for electroconvulsion, therefore leading to protection against seizures. In humans, chronic high-frequency stimulation of mesiotemporal structures has been shown to raise the seizure threshold. Velasco *et al*.[133] reported that the chronic high-frequency stimulation of the normal or the sclerotic hippocampus reduce the incidence of seizures in refractory epileptic patients without causing side effects. Patients with normal hippocampus had a 95% seizure reduction; patients with a sclerotic hippocampus had 50 to 70% seizure reduction.

### Prior findings with amygdala neurosurgery in humans

In stereotactic amygdalotomy, destructive lesions are created in both amygdalae using radiofrequency ablation for patients suffering from intractable aggression. The procedure was performed primarily in the 1960’s and the 1970’s. Narabayashi[134] introduced the stereotactic amygdalotomy in the 1960’s. Initially, the procedure was offered to patients suffering from epilepsy or EEG abnormality in addition to aggressive behavior, but eventually it was offered more broadly to patients suffering from ‘intractable aggression’. Following this initial report, several other authors reported their outcomes for the treatment of aggression and over a thousand patients have been treated[135–137]. The overall improvement in symptoms reported range between 33 and 100%, with most authors reporting 70 to 85% improvement[138]. Lee and colleagues (1998)[139] reported two patients who underwent stereotactic amygdalotomy for intractable aggression and demonstrated specifically reduced behavioral and autonomic ‘fight or flight’ activation in response to stressful cues. In these reports of stereotactic amygdalotomy, the amygdala was safely approached through a transfrontal trajectory by many investigators. Large lesions were created using wax, ethanol, or radiofrequency ablation with the goal of complete destruction of bilateral amygdala. The size of those lesions was approximately 10 to 20 times the diameter of the current DBS electrode. Despite the large lesions, in his series of 60 patients, Narabayashi *et al*.[134] observed no psychological disturbance from the procedure and only one case of transient weakness that resolved after 3 weeks. In another series, Kiloh and colleagues[140] reported seven complications following 18 operations. There were four cases of new-onset epilepsy and three cases of hypersexual behavior. The epilepsy resolved over a period of several months. Since those reports, surgical and imaging techniques have evolved considerably. Thus, no complications were reported in a more recent study where modern stereotactic and ablative techniques were used[139].

Since the advent of DBS, destructive procedures, such as subthalamotomy and thalamotomy to treat Parkinson’s disease and tremor have become obsolete. This has occurred because the safety profile of DBS is superior to destructive strategies. With minimal disruption of the cerebral tissue, the risks of hemorrhage, stroke and neurological deficits are significantly reduced. In addition, neuromodulation is reversible and side effects can often be corrected with programming changes.

There is a case report of inadvertent DBS of the main amygdala output, the stria terminalis[141]. A patient underwent the placement of bilateral globus pallidum interna (GPi) DBS electrodes (model 3387, Medtronic, MN, USA) for the treatment of dystonia. There was improvement in dystonia at the 4-month follow-up visit. Apparently soon thereafter, the left-sided electrode inadvertently migrated to the area of the stria terminalis, the major white matter tract output from the amygdala. The left electrode delivered stimulation at the following parameters: 2.7 V (amplitude), 180 μsec (Pulse width) and 180 Hz (frequency). Over the next five months, the patient developed symptoms of depression, apathy, irritability, hopelessness, and suicidality. Once the electrode was repositioned, the patient’s symptoms resolved completely. His neuropsychological condition remained unchanged from preoperative assessments through 9 total months of stimulation.

DBS of white matter tracts, like the stria terminalis, is known to cause an increase of the activity of the tract and its neuronal targets. This phenomenon commonly leads to DBS side-effects such as muscular contractions, which are due to internal capsule (white matter tract) stimulation, or diplopia, from oculomotor nerve stimulation (also white matter). In essence, DBS of the stria terminalis equates to the state of amygdala overactivity seen in PTSD patients. On the contrary, gray matter DBS has been shown clinically and by functional neuroimaging to reduce the metabolic activity of surrounding areas and neuronal targets[73]. Therefore, DBS of gray matter, such as the BLn, would likely have effects opposite those seen from DBS of the white matter in the case of the amygdala. This case is critical for several reasons:

1. It supports the model that amygdala overactivity is responsible for the symptoms of PTSD. DBS of the stria terminalis physiologically equates to amygdala overactivity. In this patient with no psychiatric history, an increase in amygdala output activity has led to symptoms commonly seen in combat PTSD (for example, irritability, helplessness, depression, and suicidality).
2. The correction of the amygdala activity led to resolution of the symptoms. As opposed to DBS of the stria terminalis, BLn DBS is expected to reduce amygdala output.
3. The patient did not suffer from a seizure or deterioration in neuropsychological status during chronic DBS for nine months. This suggests an acceptable safety profile of DBS of the amygdala.

**Figure 1:**
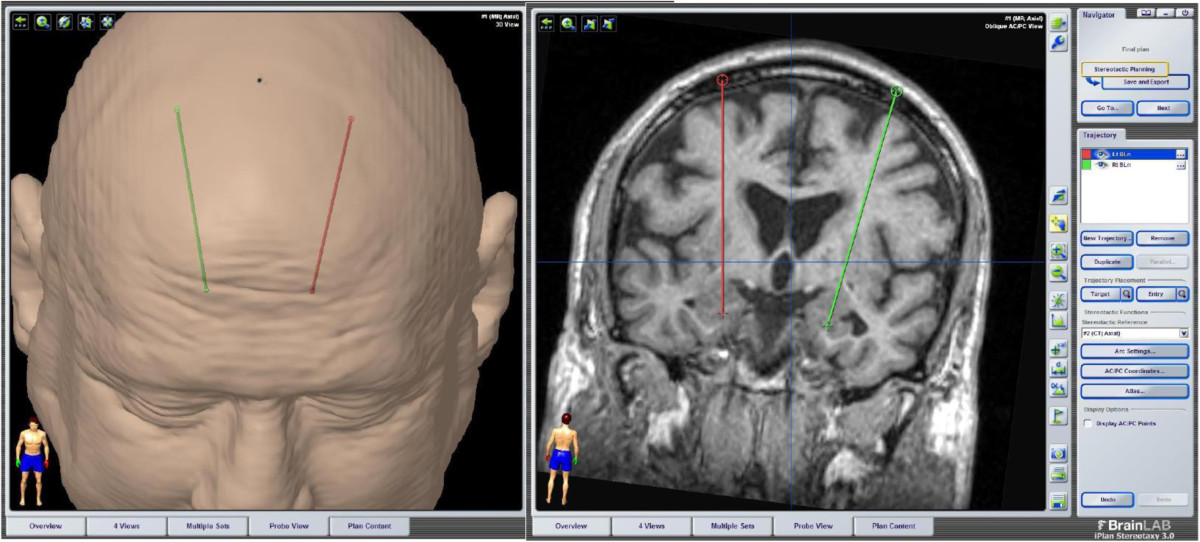
**Transfrontal trajectory for deep brain stimulation of the basolateral amygdala.**

Very recently, Sturm and colleagues[142] (2013) applied conventional DBS bilaterally to the BLn in a 13-year-old boy with intractable self-injurious behavior (SIB) in the context of mental retardation and Kanner’s autism. Over the course of 26 months follow-up at the time of publication, the authors described clinically significant improvement in SIB, as well as social communication and even language function; the child had not previously developed language and spoke for the first time beginning six months after DBS. Notably, only stimulation of the BLn, but not the central nucleus, the amygdala outflow tract, or neighboring regions affected by other contacts of the stimulating electrodes, led to benefit. Also important was the absence of significant side effects, including seizures. There was no benefit from insertion without stimulator activation, and an exacerbation occurred consequent to battery depletion with reinstatement of benefit after re-initiation of stimulation. Electrode insertion trajectory and stimulation parameters used in that single case correspond closely to those planned in the present investigation (transfrontal (Figure 1); 130 Hz, 120-μsec pulse width, 2 to 6.5-V amplitude).

Electrical stimulation of the amygdala in humans has also been performed in patients undergoing depth electrodes for epilepsy monitoring[143–145]. The amplitude delivered to the tissue was up to 10 mA, several fold higher than the recommended DBS parameters. The smaller diameter of those electrodes compared to the current DBS implant also means that the charge density delivered was several times higher than the maximum allowed by the Medtronic Activa system we will use in this study. In addition, the frequency was kept low: between 5 to 10 Hz, approximately 10 to 20 times lower than currently used for movement disorders, and 20 to 30 times lower than planned in this investigation. These settings were meant to generate seizures or other symptoms that would be related to the amygdala. The authors were intrigued that very few patients reported significant anger despite the elevated settings of stimulation. In addition, few convulsions were noted despite the high-amplitude and the fact that the patients were suffering from medically refractory temporal lobe epilepsy[143]. In our protocol, the amygdala tissue disruption and the electrical charge density are both several folds (that is, 10 to 20 times) smaller than listed in previous reports concerning amygdala neurosurgery. Therefore, we expect our safety profile to be considerably better. In addition, we are benefiting from the advances in surgical and imaging techniques since those reports were published.

Taken together, the data reviewed in the preceding sections support the proposal that persistent amygdala hyperactivity, most likely localized to the BLn, underlies the symptoms of treatment-refractory combat PTSD; a treatment that would directly reduce this hyperactivity could benefit patients whose symptoms do not respond to standard treatments. High-frequency DBS of the BLn could do this without significant risk of causing seizures or other major behavioral changes. We recognize that, as in other neuropsychiatric disorders associated with dysfunction in limbic circuitry, amygdala hyperactivation in PTSD is likely a node in an interconnected circuit, comprising at least the vmPFC, anterior insula, and extended amygdala, as well as the ventral striatum, with reciprocal connections to thalamus and hypothalamus, and output to brainstem nuclei that are also involved in the clinical phenomenology seen in the condition. While we thus agree with other investigators[146] that the BLn is not the *only* potentially valuable target for DBS in PTSD, we believe, based on the rationale described above, that it is the optimal target and it can be modulated safely with current technology.

## Methods/Design

### Purpose and justification of the study

The purpose of the current study is to evaluate the safety and potential therapeutic benefit of the Medtronic Activa DBS system implanted bilaterally in the BLn of the amygdalae in combat veterans with severe, chronic, treatment-resistant PTSD. DBS is considered a safe, nondestructive, and reversible therapy for neurological and psychiatric conditions. Despite being safe, DBS carries risks related to the surgery as well as risks associated with the neuromodulation. We expect the rate of complications from the amygdala BLn implantation procedure to be comparable to reported risks in other DBS applications, as reviewed elsewhere[147] and as detailed below.

Characterizing potentially undesirable effects of chronic high-frequency DBS of the amygdala is a principal aim of the study. While we expect there to be a low risk of serious adverse effects, the study involves extensive monitoring for psychiatric, neurologic, and neuropsychological adverse effects as described below and listed in Additional file1.

The duration of the investigation will be 24 months for each subject following device implantation and randomization. The subjects will be followed biannually once they exit the protocol. The presence of a significant other engaged in the healthcare of the patient will assist in early detection of adverse effects, as well as evaluating functional benefits.

We believe the risks involved, and the burden of extensive repeated assessments, are justified because treatment-resistant PTSD is a very serious illness associated with significant suffering and morbidity. There are currently no good treatment options for patients who have failed to improve with psychotherapy and several lines of psychopharmacological agents. We believe that BLn DBS is a therapeutic option worth investigating.

### Design of the study

This proof-of-concept trial will follow a randomized and blinded staggered-onset design for the initial three months after implantation, followed by open-label active stimulation in all subjects for a total of 24 months after implantation, with systematic monitoring of safety and efficacy. This design has been used in other recent successful early clinical proof-of-concept trials of DBS in psychiatric conditions[148]. The staggered onset of active stimulation permits assessment of the possible effect of electrode insertions without stimulation (‘microlesioning’) of the amygdala target. We expect greater improvement in PTSD symptoms in patients with initial active DBS, compared with sham stimulation, and improvement in the subgroup initially randomized to sham after stimulators are subsequently activated. The comparison of sham to active stimulation also permits a direct comparison of possible adverse effects of BLn DBS to insertion of inactive electrodes. We feel that the 2-month delay in receiving active treatment for those subjects randomized to initial sham stimulation is justified based on the scientific value of the comparisons noted, given the previously untested nature of the intervention. For ethical and investigative reasons, all subjects will receive active stimulation beginning at three months after implantation. In both treatment-refractory OCD[72] and depression[81], the benefits of stimulation increased over a 1- to 2-year period after initiation, leading us to design this pilot study with a 2-year investigational period after implantation. Given the chronic nature of illness in the subjects to be included in this study, it also makes sense heuristically to expect optimal benefit to take up to 2 years to be manifest. Equally important is the potential for adverse effects of long-term amygdala DBS to build or change over time.

The study will be conducted at the VA Greater Los Angeles Healthcare System (GLA) facilities. Several key elements make GLA the optimal location to conduct this rigorous trial and insure the safety of the subjects: 1) GLA is one of only six VA Parkinson’s Disease Research, Education and Clinical Care (PADRECC) centers. As such, GLA routinely conducts DBS procedures for the treatment of movement disorders. In addition, the GLA staff has extensive experience with the conduct of human trials in DBS. They have successfully participated in several DBS trials, including the cooperative studies for Parkinson’s disease[149] and the recent SANTE trial for the treatment of epilepsy[150]. 2) GLA treats a large PTSD population and has state-of-the-art psychiatric treatment programs for combat PTSD. The staff has extensive experience with the treatment of complex chronic PTSD in an academically affiliated medical center (UCLA) with a variety of outpatient programs, including PTSD specialty clinics, outpatient mental health centers in six to eight different communities in the greater Los Angeles area, pain clinics, post-deployment clinics, and a domiciliary. Algorithms in place allow for the safe and efficient treatment of acute psychological deterioration or transient worsening, including inpatient psychiatry and neurosurgical services. 3) GLA is also one of the 16 VA Epilepsy Centers of Excellence (ECoE). As such, it possesses unique professional expertise and advanced telemetry equipment for the monitoring, treatment, and prevention of seizures and epilepsy. In addition, the team has extensive experience with telemetry-related research.

This is a pilot study and thus will involve only six subjects, two sets of three randomized to either staggered onset time of initiation of stimulation. We believe this subject number will adequately address the proof of concept purpose of the study and provide meaningful information about the difference between active and sham stimulation (see above).

The study will provide close follow-up of patients after DBS surgery by a team involving a functional neurosurgeon, psychiatrists, an epilepsy specialist neurologist, a neuropsychologist, a geriatric neuropsychiatrist, functional neuroimaging experts, and a clinical neurophysiologist, all with expertise in psychiatric neuroscience, clinical care of veterans with PTSD, and the use of DBS. Similar to other recent studies[73, 75, 151] and guided by consensus recommendations[152, 153], we intend to publish de-identified complete details of individual patient demographic and clinical descriptors, study procedures and results, neuroimaging procedures, and DBS implantation procedures and stimulation parameters.

### Participants

The study has been approved by the GLA Institutional Review Board (IRB; PCC# 2014 -020159 2-12-14). An independent Data Safety Monitoring Board (DSMB), to which data on each subject will be submitted for review every three months for the first twelve months and then every six months for the last twelve months, has been established. The DSMB has already stipulated that the first subject be monitored for at least six months before the second subject is enrolled, after which, should there be no serious safety concerns, parallel enrollment will be permitted. The study is recruiting subjects, and has been registered at http://www.clinicaltrials.gov (PCC# 121657).

Potential subjects are recruited for the study via distribution of flyers to staff at GLA Mental Health Outpatient programs. Flyers include summarized inclusion and exclusion criteria. Potential subjects are informed about the study by their treating clinician, and given contact information for the study psychiatrist and neurosurgeon if they are interested. Charts of potential patients are then reviewed by investigators based on an IRB-issued Waiver of Consent for Screening. Patients meeting chart-review-based inclusion and exclusion criteria are invited to attend an in person assessment for further determination of eligibility. Eligible subjects will sign informed consent. After this, they will undergo baseline evaluations spaced over a six-week period, including neuropsychological testing and a baseline ^18^FDG PET scan (described below). Patients are reassessed with the Clinician Administered PTSD Rating Scale (CAPS[154]) at the end of this baseline period, and only those who maintain a total CAPS score ≥85 and other inclusion and exclusion criteria will be kept in the study. An additional baseline evaluation is stipulated by California Law referring to ‘Psychosurgery’ (WIC Sec 5326.6): all potential subjects must undergo examination by an independent team, consisting of neurosurgeons and psychiatrists uninvolved in the treatment or the study protocol, to ascertain capacity to consent, severity of illness, and inadequacy of response to standard treatments. This team of independent experts is assigned by the Medical Director of the Los Angeles County Department of Mental Health. Concomitant psychotropic medication regimens will be kept constant for the first six months of the study, unless medication-related adverse events, intercurrent medical conditions, or changes in psychiatric status make alterations clinically necessary.

### Inclusion criteria

Inclusion criteria include the following:

1. Male aged 25 to 70 years.
2. Able to give informed consent in accordance with institutional policies and participate in the 2-year follow-up, involving assessments and stimulator adjustments.
3. Patients must be stable on their current psychotropic medication for a period of 2 months before implantation and agree to not increase dosages or add any new medications for the first 6 months of the study, unless medically necessary.
4. Chart diagnosis of chronic and treatment-refractory PTSD as the principal psychiatric diagnosis and cause of distress and social/occupational impairment.
5. Confirmation of PTSD as the primary psychiatric diagnosis by the study psychiatrist via clinical interview and Clinician Administered Post-traumatic Stress Disorder Scale (CAPS) [154].
6. Confirmation of combat trauma exposure via military record review and a Combat Exposure Scale (CES) [155] score ≥9.
7. Minimum of 5-year total illness duration, with no 6-month period of clinical remission during the 5 years prior to entry in the study.
8. Clinical record documentation of nonresponse to at least 2 of the following antidepressants, alone or in combination, at maximally tolerated FDA recommended doses for ≥6 months: sertraline, paroxetine, fluoxetine, citalopram, escitalopram, amitriptyline, imipramine, nortriptyline, desipramine, clomipramine, phenelzine, tranylcypromine, venlafaxine, mirtazapine. Antidepressant trials must include at least one SSRI and one SNRI or TCA at maximally tolerated FDA recommended doses for a minimum of 3 months.
9. A minimum 3 month trial of prazosin at 10 mg per day or, if less, maximally tolerated FDA recommended doses, unless considered contraindicated based on co-morbid medical conditions or concomitant medications.
10. Trial of at least 3 months of one of the following: lithium, divalproex sodium, carbamazepine, lamotrigine, olanzapine, risperidone, bupropion either alone or in conjunction with one or more of the agents in #8 and # 9 above.
11. Six months of continuous individual psychotherapy, conducted at least twice monthly for minimum 45 minute sessions, and consisting of a) clinician-defined cognitive-behavioral psychotherapy directed toward reducing conditioned fear symptoms of PTSD; b) cognitive processing psychotherapy for PTSD [39]; c) prolonged exposure therapy for PTSD (imaginal, *in vivo*, and/or virtual reality) [47, 50]; or d) Eye movement desensitization and reprocessing therapy for PTSD including a trauma exposure component [40], with chart documentation of inadequate benefit despite concerted effort. Other forms of individual or group psychotherapy are permitted but not required for inclusion. (Patients who are unable to complete 6 months of psychotherapy may be included if the cause of treatment cessation was that the risks of further treatment, including intense psychological suffering, outweighed the potential benefits of continuing the treatment).
12. All evidence based psychotherapy for PTSD (cognitive behavioral, cognitive processing, prolonged exposure, and eye movement desensitization) has been completed a minimum of 3 months prior to enrollment.
13. Minimum baseline CAPS_17_ of 85 at entry, with a) scores of at least 4 (combined frequency and severity) on at least one symptom from each cluster (intrusion, avoidance and hyperarousal); b) score of 5 or more on CAPS_17_ items 4 or 5 (intense psychological distress or physiological reactivity on exposure to a reminder of the traumatic event); and c) no questionable validity (QV) rating greater than 1 on any CAPS item.
14. Clinically significant impairment in occupational functioning due to PTSD, manifested by one or more of the following: a) Total federal (service connected ≥70%), or State (SSI) disability compensation for at least the past 2 years for PTSD; b) global assessment of functioning score ≤45; c) no period of full time gainful employment ≥3 months in the past 5 years.
15. Clinically significant impairment in social functioning due to PTSD, manifested by one or more of the following: a) little or no social activity outside the household other than as necessary for medical appointments, practical matters such as grocery shopping, or to interact with other veterans; b) reliable description by a spouse or significant other, living with the patient, of repeated avoidance/refusal to participate in customary social engagements with friends, family or for recreational activities due to PTSD; c) two or more verbal or physical interpersonal altercations within the past year requiring another person’s intervention to prevent further escalation, or involving law enforcement
16. Cohabitation with a spouse or significant other adult person who a) can confirm the symptoms and impairment from PTSD and lack of significant symptomatic remission in the past 5 years; and b) is willing to participate with the study psychiatrist in answering questions for the life functioning in PTSD scale (LFIPS) at scheduled follow-up visits; and c) is willing to report unexpected adverse neurological or psychiatric events to study investigators and if advised by study investigators, assist the patient in accessing necessary services to address these.
17. Willingness to have unexpected neurological or psychiatric symptom shared with the study psychiatrists and other study clinicians.
18. Other medical conditions must be stable for at least 1 year, (conditions that require intermittent use of steroids or chemotherapy are excluded).

### Exclusion criteria

Exclusion criteria include the following:

1. Suicide attempt in the last 2 years and/or presence of a suicide plan (an answer of ‘Yes’ to Question C4 in Section C-Suicidality of MINI International Neuropsychiatric Interview) [156].
2. Psychosis or bipolar disorder; significant acute or ongoing risk for violence.
3. Patients primarily diagnosed with DSM-IV-TR Axis I disorder other than PTSD as determined by the MINI.
4. Within the 3 months prior to enrollment, subject has started a new psychotherapy program.
5. Alcohol or illicit substance use disorder within the last 6 months, unstable remission of substance abuse, or chart evidence that comorbid substance use disorder could account for lack of treatment response.
6. Current significant neurological conditions, including epilepsy, stroke, movement disorder; history of serious head injury with loss of consciousness.
7. Patients with uncontrolled medical conditions (such as hypertension, diabetes, and infection).
8. Uncontrolled chronic pain.
9. Baseline Montgomery Asberg Depression Rating Scale (MADRS) [157] of ≥28.
10. Patients who are receiving anticoagulation therapy.
11. Significant abnormality on preoperative structural brain MRI.
12. Electroconvulsive therapy (ECT) in the past 6 months.
13. Contraindications to MRIs or the need for recurrent body MRIs.
14. Immunosuppression.
15. Patients who are not appropriate candidates for general anesthesia and/or DBS surgery.
16. Current pursuit of new or increased disability compensation for PTSD.
17. Has cardiac pacemaker/defibrillator, implanted medication pump, intracardiac lines, any intracranial implants (aneurysm clip, shunt, cochlear implant, electrodes) or other implanted stimulator.
18. Patient has had past cranial neurosurgery.
19. Patient unable to discontinue therapeutic diathermy.
20. Use of other investigational drugs or psychotropic herbs within 30 days of baseline.
21. Patients suffering from a neurovascular condition or other intracranial process.
22. Patients suffering from a condition associated with a significant cognitive impairment.

An inclusion criterion perhaps somewhat unique to this study is the required participation of a cohabiting significant other for the duration of the study. The purpose of this aspect of the trial is first for safety purposes. We know that electrical stimulation of the amygdala has the potential to cause changes in emotional, perceptual, and behavioral functioning that subjects may not be completely aware of, be able to describe, or be able to remember. Given that this is the first attempt to target the amygdala with long-term stimulation in patients with PTSD, we felt it important to include an observer who knows the subject and is with them most of the time to increase sensitivity for detecting significant, unexpected adverse psychological or behavioral effects. To further explain this role to the significant other, an information sheet will be provided that details specific symptoms to look for, along with urgency of reporting and contact information of the investigators as well as emergency resources. Second, we believe from our own clinical experience that in many combat veterans, PTSD often affects interpersonal, family, and relationship functioning in ways that are perceived differently by veterans and their family members. Thus, we have constructed a scale for monitoring changes in interpersonal, social, and family functioning that permits assessment of patient functioning from the perspective of the patient and caregiver independently: the Life Functioning in PTSD Scale (LFIPS) [see Additional file2]. No data concerning the significant other will be collected. Furthermore, the loss of the significant other during the trial will not lead to the exclusion of the subject.

### Baseline assessments

The following baseline assessments will be completed over an extended 6-week baseline period after patients have given informed consent for inclusion, but before deep brain stimulator implantation:

1. demographics,
2. medical history (includes family history),
3. medication history,
4. physical exam,
5. neurological exam,
6. lab screening including urine toxicology,
7. 12 lead EKG,
8. MRI scan (to exclude brain abnormalities),
9. Mini International Neuropsychiatric Interview (MINI),
10. Combat Exposure Scale (CES),
11. Clinician Administered PTSD Scale (CAPS),
12. Hamilton Anxiety Rating Scale (HAM-A),
13. Montgomery-Asberg Depression Rating Scale (MADRS),
14. Young Mania Rating Scale (YMRS),
15. Global Assessment of Functioning (GAF),
16. Clinical Global Impression of Severity & Improvement (CGI-S, CGI-I),
17. Davidson Trauma Scale (DTS),
18. Columbia-Suicide Severity Rating Scale (C-SSRS),
19. Veterans Quality of Life Assessment (SF-36v),
20. Life Function in PTSD (LFIPS) (Additional file 2),
21. fluorodeoxyglucose (FDG) positron emission tomographic (PET) brain imaging,
22. Amygdala DBS in Post-traumatic Stress disorder Scale (ADIPS) neuropsychological tests (Additional file 3)
23. neuropsychological battery (Additional file 4), and
24. electroencephalogram (EEG), 30 min awake and sleep.

### Randomization and blinding

There will be two study groups, A and B, corresponding to either onset of active stimulation at 30 days or 90 days post-implantation. Prior to enrollment of the first patient, a computer algorithm will randomize each of the six potential subjects to either group A or B (three each), in blocks of two. After the 1-month post-implantation EEG telemetry session, the study neurophysiologist will learn the group assignment of each subject. Subjects and all other investigators will be blind to group assignment until completion of the trial.

### Outcome measures

The following measures will be used to assess symptomatic and functional outcomes [see Additional file1 for detailed description of measures 1 to 4]:

1. PTSD
  - Clinician Administered PTSD Scale (CAPS) [154]
  - Davidson Trauma Scale (DTS) [158]
2. Other psychiatric symptomology/morbidity
  - Montgomery-Asberg Depression Rating Scale (MADRS) [157]
  - Young Mania Rating Scale (YMRS) [159]
  - Columbia-Suicide Severity Rating Scale (C-SSRS) [160]
  - Hamilton Anxiety Rating Scale (HARS) [161]
3. Functioning/quality of life
  - Global Assessment of Functioning (GAF) [162]
  - Veterans Quality of Life Assessment (SF-36 V) [163]
  - Life Function in PTSD (LFIPS) [see Additional file 2]
4. Global outcome
  - Clinical Global Impression of Severity and Improvement (CGI-S & CGI-I) [164]
5. Neuropsychological measures
  - Amygdala Deep Brain Stimulation in PTSD Scale (ADIPS) [see Additional file 3]. Given that amygdala DBS has not previously been performed in humans with PTSD, we will also administer a unique neuropsychological battery, the Amygdala DBS in PTSD Scale (ADIPS), which was developed for this study. This battery is designed to systematically assess effects of electrode insertion and stimulation on cognitive, perceptual, emotional, and behavioral functions known to be influenced by the amygdala and its connections.
  - Neuropsychological Battery [see Additional file 4]. This battery consists of standardized measures of attention, concentration, mental effort, verbal and nonverbal memory, language, visuospatial abilities, and executive skills. It will be administered at baseline and at 6, 12, and 24 months postimplantation by a certified neuropsychologist (RM).
6. Functional neuroimaging. Preoperatively, patients will undergo a provocative PET-CT scan using fluorodeoxyglucose F-18 (FDG) to determine the baseline activity of the amygdalae, insulae, cingulate and mPFC regions as in previous studies [109, 110]. The PET procedure will include both a resting baseline study, and a second ‘stimulus provocation study’, during which patients will listen to an audio recording of a ‘trauma script’ made prior to the scanning session. The script will consist of the patient’s recitation of a narrative of the trauma that caused PTSD as in prolonged exposure therapy (for example, [47]). Study Psychiatrists (RK, HS) will be present during the PET session to ‘titrate’ symptom severity to moderate, but not intolerable levels of arousal. The same ^18^FDG PET scan paradigm will be repeated at 15 months postoperatively with optimal DBS programming parameters. Since patients enrolled in this study have failed psychotherapy, including exposure-based therapy, we expect that changes in amygdala activation observed on the second PET scan will not be the result of therapeutic habituation from the first exposure session, but rather the effect of DBS. We will compare ^18^FDG PET metabolism in the target regions between resting and stimulus-exposed conditions, and differences from preoperative to approximately one year after chronic DBS. Our data analysis of the PET scans will follow the described theory of parametric mapping using SPM (Wellcome Trust Centre for Neuroimaging) [165]. With this software, the PET data in each voxel will be normalized and fitted in a linear statistical model. Following the technique described by Shin and others [102], our hypotheses will be evaluated as contrasts where the linear compounds of the model parameters will be evaluated with t-tests. This data is then transformed into a z-score. Since we have strong directional hypotheses (that DBS reduces amygdala hyperactivity in PTSD patients), we will consider z >3.09 (*P* <0.001 one tail, cluster level) as a statistically significant difference. In this study, MM and DS will conduct and analyze the PET scans.
7. Electroencephalographic changes. After the one-month post-implantation EEG telemetry session, patients will have routine EEGs performed monthly for 15 months, and then every three months for the last nine months of the study. These will be analyzed by our study neurologist, JC, with particular attention paid to development of epileptiform changes.

Study assessments will occur weekly for five months after implantation, monthly for ten months, then every three months for the final nine months. All study measures will be completed at baseline, and then repeated as follows (see Additional files1,2,3 and4 for detailed descriptions, and Additional file5 for overall study timeline):

1. Clinical monitoring and system status check at each visit.
2. Stimulator adjustments, CAPS, MADRS, YMRS, C-SSRS, LFIPS, ADIPS, CGI, vital signs, and EEG monthly for 15 months, then every three months for nine months.
3. GAF, SF-36 V, DTS, HAMA, ECG every three months
4. Neuropsychological test battery at 6, 12, and 24 months
5. Resting and trauma script provoked PET Scan at 15 months

### Surgery and the deep brain stimulation system

The DBS system to be used in this study is the Activa PC pulse generator, a dual-channel programmable device. The programmable stimulation settings are stored in the device and are a specific combination of pulse width, rate, and amplitude settings. The entire system, manufactured by Medtronic, is comprised of implanted and nonimplanted components. The components used in this protocol are as follows:

1. The implantable pulse generator (one/subject) (Medtronic, Activa PC model 37601).
2. The intracranial leads (two/subject) (Medtronic, model 3387). The leads contain the electrodes that deliver the stimulation.
3. The lead extension wires (two/subject) (Medtronic, model 37085). These make the connection between the leads and the pulse generator.
4. Stimloc burrhole cover. This component is used to secure the lead to the skull and cover the burrhole.
5. The N’Vision clinician programmer (Medtronic, model 8840). This controls the pulse being generated.

In this trial, we will use the device as it comes from the manufacturer. The surgical implantation of the device follows the same technique used for movement disorder surgeries. The stimulation parameters that will be used in this protocol are commonly used in movement disorder and therefore the energy demand on the device will be the same as typically used in movement disorders.Following the standard surgical procedure for DBS system implantation, the patients will undergo the placement of a Leksell (Elekta, Atlanta, GA, USA) stereotactic frame. The electrode trajectory will be planned using stereotactic software (BrainLab, Munich, Germany). We will follow a traditional transfrontal trajectory (Figure 1). The transfrontal trajectory to the amygdala is a well-documented procedure traditionally used for stereotactic amygdalotomy. We will implant the DBS electrodes bilaterally in the BLn. A curvilinear incision will be made bilaterally 3 cm lateral to midline and 1 cm in front of the coronal suture. A burr hole will be placed bilaterally with a high-speed drill for placement of a Stimloc device (Medtronic, Minneapolis, MN, USA) to hold the electrode in place. Once the Stimloc is attached with the two self-tapping screws, the dura will be opened. The dura will be coagulated back and the cortex exposed, coagulated, and pierced. The electrode 3387 (Medtronic, Minneapolis, MN, USA) will then be introduced down the cannula. All the contacts of the electrode will then be stimulated at incremental voltages up to 7 volts to confirm the absence of immediate side effects by the study neurophysiologist (SK) while the patient is awake and is being examined by the study psychiatrist (RK, HS) and neurosurgeon (JPL). The left electrode will be inserted and tested first for side-effects, and then, the right electrode will be inserted and tested. In the absence of serious adverse effects, the stimulators will be turned off and the electrodes will then be tunneled under the skin down to the upper chest area. They will be connected to a dual-channel pulse generator Activa PC (Medtronic, Minneapolis, USA).

After surgery, patients will be kept in the hospital for approximately 3 days to ensure postoperative safety/stability. Then patients will return to the clinic for weekly safety evaluations for the first five months. Stimulators will be kept off in all patients for the first four weeks. Then, once the EEG telemetry session described below is complete, patients will be assigned randomly, in a double-blind fashion (only the study neurophysiologist will know) to have stimulation initiated at that 4-week postoperative time point, or after an additional 2 months (that is, 3 months postoperatively).

### Electroencephalogram telemetry session and deep brain stimulation initiation

At week 3 or 4, before initiation of experimental stimulation, each subject will be admitted to the Neurology EEG Telemetry Unit under the direction of JC for testing of stimulus parameters with real-time EEG to rule out after-discharges that would place patients at high risk of seizures, changes in vital signs that would place patients at risk of adverse health outcomes, changes in cardiac rhythm on electrocardiogram (ECG), or changes in behavior that would pose significant safety risk if they were to occur at home in an unsupervised setting. During the telemetry session, the electrodes will be initially stimulated at 2.5 V, 120-μsec pulse width, and 160-Hz frequency. The amplitude will then be progressively increased slowly to a maximum of 7 V. The pulse width will then be increased to a maximum of 210 μsec. Finally, the frequency will be increased to a maximum of 200 Hz. Side effects will be specifically noted throughout the session. If a serious side effect is noted, the last parameter changed will be kept at a maximum of 60% of the threshold value during subsequent long-term stimulation, and the other parameters will not be increased. For instance, if an after-discharge is noted for the first time at 5 V, the patient will continue in the study following our protocol, but the stimulation parameters will be kept below 3 V.

After the telemetry session, the DBS treatment will be initiated in a staggered fashion. Following the operation, patients will be randomized into two groups of three subjects. The first group will undergo therapeutic stimulation beginning 30 days postoperatively. The second group will start the stimulation at 90 days postoperatively. All the patients will undergo programming sessions of the same duration whether actual stimulation is provided or not, and the patients, the neurosurgeon, and the psychiatrists performing the evaluations will be kept blind to the treatment assignment.

We will also perform monthly EEGs for early detection of newly developed epileptiform discharges after initiation of stimulation. Subjects with newly developed epileptiform discharges will be terminated from the study and managed clinically.

### Deep brain stimulation programming

Following the initiation of stimulation, stimulation parameters will be adjusted based upon the patient’s CAPS scores and other aspects of response to DBS therapy. Only one parameter can be changed at a study visit. The neurophysiologist performing the programming will be provided with the CAPS score and will adjust the parameters, based on the following protocol (sham adjustments will be made during the first 2 months in subjects who are randomized to initiation of stimulation at 90 days post-implantation):

1. amplitude increase,
2. amplitude increase,
3. pulse width increase, then
4. frequency change.

Any further programming changes will be based on the patient’s CAPS scores, the response to DBS therapy, and the clinical judgment of the study team. In order to prevent any damage to the neural tissue, we will keep the charge-density threshold below 30 microcoulombs/cm^2^/phase. The programming device automatically provides measurement warning if this threshold is exceeded. Following every change, the patients will be monitored for any side effects. The parameters will be reverted if the subject experiences any sustained side effects.

### Patient care following the completion of the study

Patients enrolled in the study will be followed at least on a biannual basis indefinitely by the study psychiatrist and/or the study neurosurgeon after the completion of the study. In addition, the patient will continue to follow up with his treating psychiatrist. The frequency of the visits will be adjusted on the basis of the clinical care needs. All patients completing the protocol will be asked in a future IRB submission to provide long-term follow-up assessment data for publication to contribute to the literature on DBS for psychiatric disorders.

### Implant replacement

The study is budgeted for the replacement of one pulse generator per subject. Following the study, the components of the implant will be replaced indefinitely based on clinical needs. The cost will be incurred by the clinical budget of the VA since this will constitute clinical care of the patient. If FDA approval is granted for the indication and the patient wishes to continue the therapy, a depleted pulse generator will be replaced in accordance with the FDA-approved label. If FDA approval is not granted or if the application falls outside the FDA-approved label, a decision will be made based on the clinical needs of the patient. If the treating psychiatrist determines that a replacement is in the best interest of the patient, a new implant will be offered. If, after informed consent, the patient agrees with the procedure, the generator will be replaced as an off-label use of a commercially available device. The device (Activa DBS, Medtronic Inc) described in this protocol has been commercially available since 1997. There are no modifications brought to the implant for the application in PTSD; it is used as manufactured and delivered. In our routine clinical practice at GLA, several patients have undergone the placement of this implant for off-label use. This occurs primarily in the treatment of intractable pain syndrome or of movement disorders that are not described in the current FDA-approved labels. In these cases, the clinicians offer the off-label therapy to patients that have never undergone DBS. Under the current research protocol, the clinicians will have the added benefit of knowing the patient’s response to the therapy prior to offering an off-label replacement of the generator. The generator replacement is a lower-risk procedure performed under local anesthesia.

### Data analysis

This pilot study involves a small number of patients and no formal treatment hypothesis will be tested. Demographics of the patients along with baseline data and clinical information will be recorded and reported. Assessment of psychological scales and neuropsychiatric tests will be conducted according to the recommended methodology. We will compare the patients’ psychological scale scores with their baseline scores mainly for safety reasons and to ensure that our subjects are not worsening. To that end, the primary outcome measure will be the CAPS at 12 months follow-up compared to baseline. We will also analyze responders versus nonresponders to determine if any predictors can be identified. For this purpose, a clinical response will be defined as a 30% reduction in CAPS[154] from baseline and a CGI-I[164] score of 1 (very much improved) or 2 (much improved).

### Study aims

The primary aim is to assess the safety and identify adverse events of BLn DBS for treatment-resistant PTSD. Safety assessments include the following:

1. Frequency and severity of all adverse events including physiological, neurological and psychological/neuropsychological (see below).
2. Occurrence of adverse events in relation to DBS amplitude, frequency and pulse width.
3. Occurrence of electrophysiological events in relation to DBS amplitude, frequency and pulse width.
4. Electroencephalographic changes over time.

The secondary aim is to assess the effect of BLn DBS in treatment-resistant PTSD on psychiatric symptoms and quality of life using measures detailed below. A reduction of PTSD symptoms, as evidenced by the CAPS score, with a 30% reduction from baseline is defined as a response. For this study, the CAPS for DSM-IV will be used, as the protocol was developed before publication of the DSM-5[166] or the CAPS for DSM-5. The effects of BLn DBS on other psychiatric and neuropsychiatric functions will be assessed during the course of stimulation, and changes in either beneficial or adverse directions recorded and tabulated.

The third study aim will be to assess changes in brain metabolism by comparing the ^18^FDG PET scan obtained before implantation to that obtained 15 months post-implantation. Regions of interest will be the bilateral amygdalae, insulae, anterior cingulate gyri (and subregions), and other portions of vmPFC.

### Adverse events monitoring

A serious adverse event (SAE) is defined by any of the following:

1. results in death,
2. is life threatening,
3. requires hospitalization or prolongs existing hospitalization,
4. results in significant disability/incapacity, malignancy, or
5. requires an intervention to prevent impairment.

A nonserious AE is an event other than one described above. Both anticipated (that is, listed in the informed consent form) and unanticipated AEs will be recorded.

The study includes an independent Data Safety Monitoring Board appointed by the IRB at our institution. Once an AE is identified, the primary concern will be to attend the subject and address the problem and ensure safety. Immediately upon discovery of an SAE, the primary investigator will inform the DSMB, the IRB, and the FDA. The IRB and the DSMB will also be promptly informed of the occurrence of any AEs via review of case report forms completed at each visit for all included subjects.

### Anticipated adverse events, complications, and side effects

#### Surgery

Patients will be informed that implantation of a DBS system involves the following risks: postoperative pain, stress, or discomfort; intracranial hemorrhage; subcutaneous hemorrhage or seroma; infection; seizure or convulsions; aphasia; cranial neuropathy; amnesia; paralysis; stroke; death; cerebrospinal fluid leakage; additional neurosurgical procedure to manage one of the above complications or to replace a fractured lead; excoriation of the implant; and additional surgical procedure to replace the pulse generator.

#### Amygdala stimulation

More specific to this study, anticipated adverse events may be subdivided into five areas:

1. Emotional - happiness, even mania, sense of calm, anxiety, fear, anger, depression, sense of doom, or suicidal thoughts. Lack of anxiety or even lack of normal fear reactions to potentially dangerous situations are possible side effects.
2. Sensory and perceptual - seeing, hearing, feeling, smelling, or tasting things that are not there; feeling as if you are separate from your body; déjà vu (feeling as if a place that should be unfamiliar is familiar); jamais vu (the opposite of déjà vu); other changes in the way things look, sound, feel, taste, or smell; or out-of-body experiences.
3. Behavioral - increased or decreased appetite, sleep, or sexual interest; suddenly starting to run; increased or decreased interest or participation in religion, politics, moral issues, or writing; tendency to put nonfood objects in the mouth; aggressive behavior toward self or others; or suicide attempt.
4. Neuropsychological - improvement or worsening of attention, memory, language function, visual-spatial abilities, logical reasoning, risk-taking, or ability to understand emotions or other psychological issues in others.
5. Neurological (seizures) - risk of seizures from electrical stimulation of the amygdala. These seizures may arise as a result of the direct stimulation of the brain tissue or as a result of modifications (such as scarring or sclerosis) of the brain tissue over time.

### Risk reduction measures

#### Surgery

The surgical procedure involved in deep brain stimulation electrode placement is considered to carry a low risk. In order to further reduce the risks of complication associated with the surgery we are performing the following procedures:

1. Transfrontal trajectory. The transfrontal trajectory employed has been used in more than a thousand cases of stereotactic amygdalotomies [138]. Using this trajectory (Figure  1), the incidence of complications from the surgery is comparable to that of DBS for movement disorders.
2. Strict exclusion criteria of ‘higher risk’ patients. Those patients would carry a higher risk of complication related to the use of anticoagulation or the presence of a medically uncontrolled condition (for example, diabetes, hypertension, or infection). By eliminating those patients, we will significantly reduce the risks of major complications.
3. Use of computer-assisted stereotactic targeting. This technique allows us to precisely predict and determine the trajectory followed by the electrode on the preoperative MRI. Using this technique, we can avoid major vessels and the ventricles. This will further reduce the risks of hemorrhage and mistargeting.
4. Intensive postoperative care. This includes the use of an immediate postoperative CT scan to rule out an intracranial complication and the admission of every patient to the intensive care unit for frequent neurological examination. These steps will permit us to promptly recognize the presence of a complication.
5. Use of perioperative antibiotics. Antibiotics will be administered one hour prior to incision and then for a period of 24 hours.

#### Neuromodulation

The side effects associated with neuromodulation are typically reversible with adjustments given the nonlesional nature of high-frequency stimulation. In order to reduce the risks associated with neuromodulation, we will perform the following procedures:

1. The protocol includes a monitoring session at the beginning of the stimulation where the patient undergoes telemetry, electrocardiography, and oximetry while the stimulation parameters are slowly increased (see above). This session will be critical to monitor for the occurrence of any afterdischarges or electrographic seizures. The risk of electroconvulsion is significantly reduced in the absence of afterdischarges. Bawden and Racine [167] reported that electrical stimulation of the amygdala with a charge density below the threshold to produce afterdischarges does not significantly lower the afterdischarge threshold in rats over time. Other authors have reported that electrical stimulation of the amygdala and the hippocampus raise the threshold for electroconvulsion therefore leading to protection against seizure. In humans, chronic high-frequency stimulation of the mesiotemporal structures has been shown to raise the seizure threshold. Velasco *et al*. [133] have reported that the chronic high-frequency stimulation of the normal or sclerotic hippocampus reduces the incidence of seizures in refractory epileptic patients without causing side effects. Patients with a normal hippocampus had a 95% seizure reduction; patients with a sclerotic hippocampus had a 50 to 70% seizure reduction.
2. The protocol includes a systematic plan for monitoring, with specified operational criteria for interventions to manage the two most serious categories of risk related to amygdala neuromodulation: seizures/epilepsy, and serious psychiatric disturbance, including suicidal or aggressive behavior. Monthly EEG surveillance will be performed to evaluate for the occurrence of epileptiform discharges.
3. DBS will be initiated at low (subtherapeutic) parameters and will be raised slowly over a period of several days.
4. During a programming session, only one parameter (amplitude, pulse width or frequency) will be increased.
5. Following an adjustment in the parameters, the patient will be kept in the clinic for at least 30 min to make sure that no acute changes related to neuromodulation occur.
6. The inclusion of patients’ significant others will allow early detection and management of adverse psychiatric or neurological effects that develop between clinic visits.
7. If side effects are occurring with new stimulation settings, the parameters will be decreased until the side-effects have resolved. This follows our current clinical programming protocol for our patients treated with DBS.
8. Subjects will be provided with an emergency identification card. The same card will also be provided to significant others participating in the study and, in addition, significant others will be provided with an information sheet that describes potential adverse psychiatric and neurological effects of DBS, and provides guidelines on how to respond.
9. Beginning not earlier than six months after implantation, patients who have experienced benefit from stimulation without treatment interfering adverse effects, and who are able to understand the nature of the devices and treatment well enough, will be offered an external controller they can use to verify the device on their own. This will permit patients to have an additional safeguard against unexpected adverse effects that emerge between visits, particularly if patients want to travel significant distances away from the medical center.

### Management of potential complications and therapy cessation

#### Seizures

Because the stimulation parameters chosen in this study will avoid the induction of afterdischarges, it is unlikely that kindled seizures will occur. However, given the possible kindling effect of chronic DBS of the amygdalae, monthly EEG studies will be performed to detect epileptiform discharges before epilepsy would be fully established. Subjects with confirmed epileptiform discharges, such as sharp waves, spike and slow waves, polyspikes, or a full seizure will not be kept in the study. The EEG will be interpreted by a board-certified clinical epileptologist (JC).

Any patients who suffer from a seizure will follow up with our epileptologist on a monthly basis for a period of six months, and then every three months for the duration of the trial. Following the completion of the trial, the patient will continue to be followed up as clinically indicated.

### Worsening psychiatric condition

The therapy aims at improving the psychiatric condition of the patient; however, our pre-specified algorithm will assist in caring for patients who may experience worsening of their condition while on therapy. The decision to discontinue a patient‘s participation in the study due to worsening symptoms will be individualized, since it is possible that some symptom or function domains will improve at the same time that others will worsen, and that some subjects will prefer to remain in the trial despite temporary worsening of their condition. For instance, patients with overall improvements may face temporary challenges related to higher expectations from their social environment (for example, employment or home responsibilities) and this could lead to deterioration on different scales. Significant worsening in psychological status will be defined as >30% worsening on standardized rating scales (and/or clinically significant change). The management will follow a predetermined algorithm.

The presence of significant suicidal/homicidal impulses will be evaluated at baseline via clinical interview, including standard VA Computerized Patient Record System templates for suicide and violence risk assessment, and the CSSRS-Baseline[160]. Subjects with more than low levels of risk for suicide or violence/homicide will be excluded from participation. During the study, study psychiatrists will conduct clinical assessment of suicidal and violent/homicidal thoughts at each visit, and questions about these risks will also be asked of significant others. Significant others will be asked to contact study investigators immediately and, if necessary, call 911 or contact the VA Suicide Hotline should these issues emerge between visits. At monthly visits for the first year, and every 3 months during the second year, the CSSRS-Since Last Visit is already included in the study protocol. Planned interventions for emergent suicidal or homicidal/violent events will be individualized based on specific findings and circumstances, but will conform to the following general algorithm:

1. New suicidal or violent thoughts without intent or plan to act:
  - identify and manage intercurrent nonstudy related medical, psychological, or social factors;
  - assess for presence of clinical syndromes such as major depression, mania, or psychosis, and intervene with psychotherapy or medications as clinically indicated; and
  - adjust DBS parameters to prior settings that were not associated with such thoughts/impulses, or turn stimulator(s) off if this occurs at the first initiation of active stimulation.
2. New suicidal or violent/homicidal thoughts with plan or intent to act, or commission of suicide attempt or violent act: immediate psychiatric hospitalization, voluntary or involuntary as per standard legal guidelines applicable to all veterans. Then, in the hospital, 1a, 1b, and 1c, are followed as above.
3. Actual completed suicide or homicide: support will be provided to the caregiver or patient and caregiver, respectively; the VA’s Suicidal Behavior and Violence Committee will be notified; the IRB, DSMB, and FDA will be notified; all other patients and significant others involved in the trial will be notified; no further enrollment will be permitted until a full assessment of the relationship of the incident to the study has been completed; and overall continuation of the trial will depend on the outcome of thorough investigation and IRB/DSMB recommendations.

### Therapy cessation

The decision to stop the stimulation unilaterally (one electrode) or bilaterally (both electrodes) will be made on a case-by-case basis and the IRB will be informed. The decision to stop the stimulation is not necessarily irreversible and could be used in certain cases to confirm that a specific symptom is not caused by the stimulation. Patients who stop the stimulation, but agree to follow-up, will be seen at the same frequency, following the protocol. As a general guideline, cases where the stimulation would be stopped include:

1. After being fully informed, the patient wishes to stop the stimulation.
2. The patient is suffering from a deterioration of his psychological condition thought to be related to the stimulation. He has been unresponsive to changes in DBS parameters.
3. The stimulation is causing intolerable side effects unresponsive to changes in DBS parameters.
4. The patient has suffered from a seizure as a result of DBS.
5. The patient has to undergo treatment for a life-threatening condition and stimulation may interfere with the treatment.
6. The patient has become seriously non-compliant with the course of therapy (for example, missing several appointments, engaging in behavior that places him or others at risk related to the device, or serious substance-use disorder).

## Discussion

Treatment-resistant PTSD, particularly among combat veterans, is a serious condition associated with substantial morbidity and likely early mortality. While there are effective treatments, particularly trauma-focused cognitive-behavioral psychotherapies, that can help many of these patients, there are individuals who do not benefit from, or tolerate, these and psychopharmacologic interventions. The rationale for amygdala DBS is well supported both from preclinical direct studies of amygdala function, and the success of BLn DBS in a rat model of PTSD by our own group. Human functional neuroimaging findings are also strongly suggestive of amygdala hyperactivity as an underlying substrate of persistent PTSD, particularly the symptoms of stimulus-associated emotional and autonomic hyperarousal. While alternative targets for neuromodulation with DBS in PTSD have been proposed[146], our model, proposing high-frequency DBS of the bilateral BLn in treatment-refractory combat veterans, has the best overall support. It is feasible both from a technical neurosurgical perspective[147] and safe based on a recent clinical report of DBS with the same target for another treatment-refractory behavioral condition[142].

The main concern is that the potential benefit of BLn DBS comes with the risks of any DBS neurosurgical procedure, as well as risks associated with long-term neuromodulation. Among the surgical risks, we foresee the risk of seizures to be theoretically greater than seen in other DBS applications in neurology and psychiatry. However, the case noted above[142], in which stimulation parameters similar to those planned for our study were not associated with seizures, and the findings of our literature review above indicating that much higher charge densities than planned for our devices are needed to trigger or kindle amygdala seizures in non-epileptic individuals, mitigate this risk. Further, our protocol includes a baseline stimulus-testing EEG telemetry session and monthly EEGs during chronic stimulation, all monitored by an epileptologist involved in our study. In terms of the risks of neuromodulation, our protocol involves extensive systematic monitoring using both validated and novel psychiatric and neuropsychiatric measures designed to identify changes in emotion, perception, thinking, behavior, and autonomic and vegetative function that could be influenced by amygdala circuitry neuromodulation. Given that this is the first trial of BLn DBS in a psychiatrically ill population, this appears justified. As noted, our study includes the fairly unique requirement that patients who enroll in the study do so in conjunction with a co-habiting significant other willing to work with the investigative team in monitoring for both foreseeable and unforeseeable safety risks. Our protocol’s emphasis on improving both function and symptoms also involves asking the significant others to provide input about changes in veterans’ social functioning that will help us better understand how amygdala neuromodulation changes PTSD and affects human behavior.

The study includes two specific elements that hope to add to the understanding of brain-behavior relationships from our intervention. First, the double-blind staggered-onset sham-stimulation phase will allow us to observe changes associated with electrode implantation without electrical neuromodulation; what others have referred to as a ‘microlesioning effect’. This will also allow us to separate out nonspecific aspects of study involvement from specific effects of DBS. The study also includes prestimulation and poststimulation PET scanning sessions, each of which includes scans done before and after exposure to a trauma narrative as has been done in other functional neuroimaging studies of PTSD. We chose to use the ^18^FDG PET paradigm because it permits assessments of change in stimulus-driven functioning of the brain for longer time epochs than either ^15^O_2_ PET or fMRI. We feel that this more closely models the clinical difficulty patients with PTSD face; it is not just the nature and intensity of reactions, but their duration and ensuing effect on functioning that is important. We considered including a pre-implantation, as well as a pre-stimulation, post-implantation PET study. While this would be ideal from a scientific perspective, we decided that the additional patient burden was not justified given the completely uncharted territory of this pilot investigation.

The principal limitation of this study protocol is the extensive burden of monitoring required of patients. Weekly visits for the first five months, and monthly thereafter for another 19 months, are required of consenting subjects. Procedures at the monthly visit include fairly extensive batteries of psychiatric and neuropsychiatric tests that - while they may be less cumbersome with repetition - could still be fairly trying. The additional requirement for frequent EEGs, two PET scans, and three neuropsychological testing batteries add additional burden. For the patients who would qualify - combat vets disabled by their illness with objectively confirmed severe symptomatology and social function impairment that has not resolved despite extensive standard treatment efforts - asking them to do this is difficult and will require intensive involvement on the part of study team. To compensate patients for the burdens of the study investigations, we have been accorded funding to pay them transportation costs, and, when necessary, overnight accommodations, including for significant others. The principal investigators have made a commitment to be available - or to provide adequate clinical coverage when away - to subjects and their significant others 24/7 for the duration of the study. An independent data safety monitoring board will provide oversight throughout the study as well.

We hope that our study will benefit the patients who participate. We also hope that BLn DBS is found to be safe in this population and that it can be extended in future studies to other populations of individuals suffering from treatment-refractory PTSD.

## Trial status

The study is currently recruiting subjects.

### Regulatory issues

The device (Activa DBS, Medtronic Inc) described in this protocol has been commercially available since 1997. In this study, the device will be used for patients suffering from PTSD, which is not an approved indication at this time; approval for use in this study is granted under IDE #G120095/S001 (Revised 5-8-14 under G120095/R002). The device does have pre-market approval for use in Parkinson’s disease and essential tremor for which over 70,000 patients have undergone implantation and subsequent treatment worldwide. DBS received Humanitarian Device Exemption for use in dystonia (HDE #020007) and obsessive-compulsive disorder (HDE #050003).

In this trial, the device and the individual components will not be modified - it will be kept in its packaging until implantation and will be used as it comes from the manufacturer. The surgical implantation of the device follows the same technique as used for movement disorder surgery

### Institutional Review Board (IRB)

IRB-A, VA Greater Los Angeles Healthcare System

11301 Wilshire Blvd., Mail Code 151

Los Angeles, CA 90073

Phone: 310-268-3345

Fax: 310-268-3774

## Authors’ information

RK is Staff Psychiatrist at the Sepulveda Ambulatory Care Center, VAGLAHS and Clinical Professor of Psychiatry and Biobehavioral Sciences, David Geffen School of Medicine at UCLA.

JL is the neurosurgeon for the Southwest PADRECC and the Southwest Epilepsy Center of Excellence at the VAGLAHS and is Assistant Professor-in-Residence in Neurosurgery, David Geffen School of Medicine at UCLA.

SK is a Clinical Neurophysiologist, Professor in the UCLA Department of Neurosurgery, and Deputy Associate Chief of Staff for Research at the VAGLAHS.

HS was a PGY-IV Psychiatry resident in the UCLA/San Fernando Valley Psychiatry Training Program and Psychiatry Consultant for the DBS Program, Department of Neurology, Cedars-Sinai Medical Center, Los Angeles at the time this manuscript was written. She is currently Research Psychiatrist, VAGLAHS (without compensation), and Attending Psychiatrist, Cedars Sinai Medical Center, Los Angeles.

HK is a research biologist with the VA Greater Los Angeles Healthcare System.

RM is a Research Psychologist and Gero/Neuropsychologist at the VAGLAHSand an Assistant Research Psychologist in the Department of Psychiatry & Biobehavioral Sciences at the David Geffen School of Medicine at UCLA.

MM is Professor of Physics at UC Irvine and Clinical Professor of Radiological Sciences. He is Director of Positron Emission Tomography at the VA Greater Los Angeles Healthcare System.

JC is Staff Neurologist at the VAGLAHS, Director of the VAGLAHS/UCLA Clinical Neurophysiology Fellowship Program, Director of the Epilepsy Center of Excellence, WLAVA and Associate Professor of Neurology at UCLA.

DS is Director of the Gero/Neuropsychiatry Division, VAGLAHS and Professor-in-Residence in the Department of Psychiatry and Biobehavioral Sciences, David Geffen School of Medicine at UCLA.

## Electronic supplementary material

Additional file 1: **Outcome Measures: Detailed description of psychiatric symptom measures, with references**[154, 157–164, 166]**.**(DOCX 18 KB)

Additional file 2: Life Functioning In PTSD Scale (LFIPS): Scale developed for this study for use in assessing impact of PTSD on family, recreational and occupational functioning.(DOCX 31 KB)

Additional file 3: **Amygdala DBS in PTSD Scale (ADIPS): Scale developed for this study that consists of three standard measures for assessing Amygdala-related psychological functions and one inventory developed for this study to monitor behavioral, emotional, personality, sensory, perceptual and neurovegetative changes with amygdala DBS**[168–170]. (DOCX 40 KB)

Additional file 4: **Neuropsychological Battery: List of standardized assessments of attention, concentration, language, memory, visuospatial and executive function to be administered at intervals during study**[171–184]. (DOCX 16 KB)

Additional file 5: Study Timeline.(DOCX 41 KB)

Below are the links to the authors’ original submitted files for images.

Authors’ original file for figure 1

## Abbreviations

ADIPS: Amygdala DBS in PTSD Scale
AE: adverse effect
SAE: serious AE
ALIC: anterior limb of internal capsule
BLn: basolateral nucleus
BOLD: blood oxygen level dependent
CAPS: Clinician Administered PTSD Scale
CES: Combat Exposure Scale
CGI-I: Clinical Global Impression of Improvement
CGI-S: Clinical Global Impression of Severity
CPT: cognitive processing therapy
CS: conditioned stimulus
C-SSRS: Columbia-Suicide Severity Rating Scale
DBS: deep brain stimulation
DSM: Diagnostic and Statistical Manual of Mental Disorders
DSMB: Data Safety Monitoring Board
DTS: Davidson Trauma Scale
ECG: electrocardiogram
ECT: electroconvulsive therapy
EEG: electroencephalogram
EMDR: eye movement desensitization and reprocessing therapy
FDA: US Food and Drug Administration
FDG: ^18^fluorodeoxyglucose
fMRI: functional magnetic resonance imaging
GAF: Global Assessment of Functioning
GLA: Veterans Administration, Greater Los Angeles Healthcare System
GPi: Globus Pallidum Interna
HAM-A: Hamilton Anxiety Rating Scale
HDE: humanitarian device exemption
IDE: investigational device exemption
IRB: Institutional Review Board
ISTSS: International Society for Traumatic Stress Studies
LFIPS: Life Function in PTSD Scale
MADRS: Montgomery Asberg Depression Rating Scale
MINI: MINI International Neuropsychiatric Interview
mPFC: medical prefrontal cortex
mTBI: mild traumatic brain injury
NCPTSD: National Center for PTSD
NIMH: National Institute of Mental Health
OCD: obsessive-compulsive disorder
OIF/OEF: Operation Iraqi Freedom/Operation Enduring Freedom
PADRECC: Parkinson’s and Related Disorders Research, Education and Clinical Center
PE: prolonged exposure therapy
PET: positron emission computed tomography
PTSD: post-traumatic stress disorder
RCT: randomized controlled trial
SANTE: stimulation of anterior nucleus of thalamus for epilepsy
SF-36v: Veterans Quality of Life Assessment
SPECT: single photon emission computed tomography
SPM: statistical parametric mapping
SSRI: serotonin selective reuptake inhibitor
TBI: traumatic brain injury
UCLA: University of California at Los Angeles
UCS: unconditioned stimulus
VA: Veterans Affairs
VARO: VA Research Office
VHIS: Vietnam Head Injury Study
vmPFC: ventromedial prefrontal cortex
WIC: Welfare and Institutions Code
YMRS: Young Mania Rating Scale.
