# Supplementary material for: Deep brain stimulation of the basolateral amygdala for treatment-refractory combat post-traumatic stress disorder (PTSD): study protocol for a pilot randomized controlled trial with blinded, staggered onset of stimulation
Source: Trials. 2014 Sep 10;15:356. doi: 10.1186/1745-6215-15-356 (PMC4168122; doi:10.1186/1745-6215-15-356)
Supplement: Supplementary file 1 — Additional file 1: Outcome Measures: Detailed description of psychiatric symptom measures, with references[154, 157–164, 166].(DOCX 18 KB) [file 13063_2014_2226_MOESM1_ESM.docx]

**Additional File 1. Outcome Measures**

CLINICIAN ADMINISTERED PTSD SCALE (CAPS) [154]

This assessment can be used to diagnose PTSD, determine if it is a current or lifetime diagnosis, and assess symptom frequency and severity over time. The CAPS consists of structured interview questions designed to assess both severity and frequency of each of the 17 symptoms of Post-Traumatic Stress Disorder that are listed in the DSM-IV criteria for PTSD (total score 0-136). It can also detect five associated features: guilt, dissociation, derealization, depersonalization, and reduction in awareness of surroundings. It also assesses the impact of the 17 PTSD symptoms on social and occupational functioning. While the diagnostic criteria for PTSD have been changed for DSM-5 [161], and a DSM-5 based CAPS interview has been published (<http://www.ptsd.va.gov/professional/assessment/adult-int/caps.asp>), this protocol was developed based on the DSM-IV CAPS.

DAVIDSON TRAUMA SCALE (DTS) [162]

The DTS is a 17 item self-rated scale with each item corresponding to the 17 DSM-V symptoms of PTSD. The severity and frequency of each symptom is rated along with the distress associated with those symptoms.

HAMILTON ANXIETY RATING SCALE (HARS) [163]

A 14 item clinician-administered scale designed to assess the severity of anxiety symptoms. All questions are rated on a 5 point (0-4) scale, with 7 questions on psychic anxiety and 7 questions on somatic anxiety. Total scores range from 0 to 56.

MONTGOMERY-ASBERG DEPRESSION RATING SCALE (MADRS) [157]

The MADRS is a clinician administered 10 item test with each item rated on a 7 point (0-6) scale to evaluate the severity of depressive symptoms in patients diagnosed with a depressive disorder. It is designed to be sensitive to change in depressive state during treatment. Total scores range from 0-52 with score interpretation of: 7 recovered, 15 mild depression, 25 moderate depression, 31 severe, and 44 very severe depression.

COLUMBIA-SUICIDE SEVERITY RATING SCALE (C-SSRS) [164]

The C-SSRS is a semi-structured clinical interview that utilizes a set of prompts and questions to help an interviewer assess the full range of suicidal ideation and behavior, and the intensity of ideation. The use of the C-SSRS in this study complies with FDA recommendations for systemic monitoring of suicidality in trials of interventions for psychiatric and/or behavioral disorders.

THE YOUNG MANIA RATING SCALE (YMRS) [165]

The YMRS is a clinician administered 11-item instrument used to assess the severity of mania in patients with a diagnosis of bipolar disorder. Total scores range from 0-52.

GLOBAL ASSESSMENT OF FUNCTIONING (GAF) [166]

The GAF is administered by qualified and trained psychiatric personnel. It is designed to rate overall psychological, social and occupational functioning, but does not include any physical or environmental impairments. The patient is rated at the end of a diagnostic interview on a single score scale of 1-100, with the scale being divided into ten ranges of functioning. We are aware that this scale is no longer used in DSM-5, and that the WHODAS is recommended as an alternative for consideration [161]. However, the study was developed and approved before DSM-5’s publication, and most clnicians are familiar enough with the GAF to make it meaningful.

VETERANS QUALITY OF LIFE ASSESSMENT (SF-36V) [167]

The Veterans SF-36 is a self-report health survey consisting of 36 questions that measures 8 aspects of physical and emotional health. The physical scales measure general health (GH), physical function (PF), role limitations due to physical health (RP) and bodily pain (BP). The mental scales measure mental health (MH), role limitations due to emotional problems (RE), energy/vitality (VT) and social functioning (SF). The Veterans’ version is comparable to the Medical Outcomes SF-36 for 6 of the 8 scales. The RP and RE scales have been modified from yes/no response choices to five-point ordinal choices.

LIFE FUNCTION IN PTSD (LFIPS) (Additional File 2)

This clinician rated scale was developed specifically for evaluating function in PTSD patients as the current literature does not contain any standardized instruments of assessing patient functioning from the perspective of a significant other. It measures six domains: Family Life, Marriage, Children, Social Interactions, Leisure, and Work and Productivity. Each question is rated on a scale of 1-5 with 5=Excellent and 1=Extremely poor. Total scores range from 18-80 with lower score indicative of poor life functioning. Both patient and significant other are assessed independently and scores compared to assess for differences.

CLINICAL GLOBAL IMPRESSION OF SEVERITY AND IMPROVEMENT (CGI-S & CGI-I) [168]

CGI is a clinician-rated scale used to assess global severity of illness and treatment response (improvement or worsening) in psychiatric patients. Both Severity of Illness and Improvement are rated on a seven-point scale with illness ranging from 1=normal to 7=extremely ill, and treatment response, 1=very much improved to 7=very much worse.
