# Supplementary material for: Deep brain stimulation of the basolateral amygdala for treatment-refractory combat post-traumatic stress disorder (PTSD): study protocol for a pilot randomized controlled trial with blinded, staggered onset of stimulation
Source: Trials. 2014 Sep 10;15:356. doi: 10.1186/1745-6215-15-356 (PMC4168122; doi:10.1186/1745-6215-15-356)
Supplement: Supplementary file 2 — Additional file 2: Life Functioning In PTSD Scale (LFIPS): Scale developed for this study for use in assessing impact of PTSD on family, recreational and occupational functioning.(DOCX 31 KB) [file 13063_2014_2226_MOESM2_ESM.docx]

| ***A. FAMILY FUNCTIONING*** | | | | | |
| --- | --- | --- | --- | --- | --- |
| 1. SOCIAL INTERACTIONS AT HOME | Initiates and engages in generally positive or at least socially appropriate ways | May or may not initiate, but engages, with mostly appropriate, but sometimes inappropriately angry or fearful responses | Rarely initiates, engages, but with mixture of appropriate and inappropriately angry or fearful responses. | No initiation, reluctant or limited engagement with often fearful or angry responses | Little or no engagement |
|  | 5 | 4 | 3 | 2 | 1 |
| 2. EATING WITH FAMILY AT HOME | Consistently joins and is generally positive | Consistently joins, but exhibits a mixture of positive and negative affects | Consistently joins, but is generally quiet, angry or anxious | Joins only inconsistently | Rarely or never sits with others for meals |
|  | 5 | 4 | 3 | 2 | 1 |
| 3. SOCIAL ENGAGEMENTS WITH FAMILY  E.g., eating out, beach, movies, birthday Party, holiday | Participates in planning, as well as engages, enjoys | May participate in planning, engages, at least sometimes without being asked, and does so either with/without enjoyment | Engages only when asked, may or may not enjoy | Engages only reluctantly, and/or with anger/disgruntlement/withdrawn responses. | Refuses to engage |
|  | 5 | 4 | 3 | 2 | 1 |
| **FAMILY TOTALS** |  |  |  |  |  |

| ***B. MARITAL/INTIMATE RELATIONSHIP FUNCTIONING*** | | | | | |
| --- | --- | --- | --- | --- | --- |
| 1. GENERAL INTERACTIONS | Consistently warm, friendly, loving and engaged; concerned about sig other’s life. | Relates amicably, but with periods of anger, aggression, irritability; moderate concern for sig. others’ life concerns. | Relates, but either indifferent or minimalistic interactions. | Avoids interactions much of the time, relates in sullen, angry, fearful or critical ways for the most part | Either doesn’t interact at all, or is angry, hostile, or irritable when he has to interact. |
|  | 5 | 4 | 3 | 2 | 1 |
| 2. COOPERATION | Enthusiastically engages in tasks or activities requested or suggested by s.o. | Engages without protest, but is sometimes positive, other times not | Typically engages, but does so with mostly quiet, passive agreement | May or may not refuse, but may or may not get angry or irritable | Typically refuses and gets angry about being asked |
|  | 5 | 4 | 3 | 2 | 1 |
| 3. INTIMACY | Consistently engages and initiates, with mutual satisfaction | Occasionally initiates; engages when approached by spouse, mutual satisfaction may or may not be consistent | Does not initiate; engages, but with minimal enthusiasm and typically not mutual satisfaction | Avoids or only reluctantly engages, without usually mutual satisfaction | Refuses, or if there is engagement, it leads to negative outcome for both parties |
|  | 5 | 4 | 3 | 2 | 1 |
| **MARITAL TOTALS** |  |  |  |  |  |

| ***C. INTERACTION WITH CHILDREN/GRANDCHILDREN*** | | | | | | |
| --- | --- | --- | --- | --- | --- | --- |
| 1. GENERAL | Loving, amiable, appropriate most of the time. Engages with them | Engages with kids, but not consistently positive emotions and interactions | | Engages only sparingly, with inconsistent mix of (+) and (-) emotions | Engages only when necessary and cold/numb, fearful, angry or irritable in interaction | Avoids engaging with kids at all, and/or they avoid him because of concerns about angry responses. |
|  | 5 | 4 | | 3 | 2 | 1 |
| 2. PLAY (MAY ALSO INVOLVE HELPING WITH HOMEWORK) | Initiates, plans and leads, with generally positive interactions and outlook | Initiates or engages, but mood alternates between positive/enthusiastic and angry/fearful/irritable | | Participates in such activities, but is “going along” and doesn’t seem to really be enthused about it. | Has to be pushed/convinced, and joins only intermittently or reluctantly and mood is generally not positive | Rarely or never participates in such activities, no matter what efforts are made to convince him. |
|  | 5 | 4 | | 3 | 2 | 1 |
| 3. DISCIPLINE | Firm but appropriate and controlled | Acts appropriately, but children are fearful because of what they perceive is possible severe anger display | | Disciplines with anger out of proportion to situation, causing discomfort in kids, but still appropriate | Acts inappropriately most of the time, but still involved in discipline because has occasions when discipline is appropriate | Anger dyscontrol such that kids are terrified and the effort is ineffective. Makes problems worse or is unable to be involved |
|  | 5 | 4 | | 3 | 2 | 1 |
| **CHILDREN TOTAL** |  | |  |  |  |  |

| ***D. SOCIALIZATION/FRIENDSHIPS (OUTSIDE FAMILY)*** | | | | | |
| --- | --- | --- | --- | --- | --- |
| 1. SOCIAL INTERACTIONS WITH FRIENDS | Initiates contact, engages, interacts and enjoys | Doesn’t initiate engagement, but accepts invitations and engages and interacts and enjoys | Engages when asked, but little interaction or enjoyment | Refuses to get together with friends when asked, or only if he can’t avoid it. | No interaction, or only interactions that are filled with fear or anger |
|  | 5 | 4 | 3 | 2 | 1 |
| 2. SOCIAL INTERACTIONS WITH STRANGERS | Interacts without conflicts/problems | Interacts with occasionally excessive negative emotional reactions | Mixture of appropriate and negative emotional responses during interactions | Minimal interactions, and/or usually characterized by negative emotional reactions | No interactions, or only fear or anger. Any incident of physical violence toward property or others. |
|  | 5 | 4 | 3 | 2 | 1 |
| **FRIENDSHIPS TOTAL** |  |  |  |  |  |

| ***E. LEISURE ACTIVITIES*** | | | | | | | |
| --- | --- | --- | --- | --- | --- | --- | --- |
| HOBBIES/RECREATIONAL INTERESTS | Regularly engages with enjoyment/satisfaction | Engages with significant frequency, but with reduced satisfaction compared with when well | | May engage, but less frequent than when well, and/or with only limited enthusiasm | Rarely engages, and there is little enjoyment | | No active engagement |
|  | 5 | 4 | | 3 | 2 | | 1 |
| **LEISURE SCORE** |  | |  |  | |  |  |

| ***F. OCCUPATIONAL FUNCTIONING*** | | | | | |
| --- | --- | --- | --- | --- | --- |
| VA Service Connected? No_____ Yes _____, _______% due to PTSD; ______% due to ________________  Social Security Disability? No _______ Yes _______, Due to ______________________________________  Other Disability No ______ Yes ________, Due to _______________________________________________ | | | | | |
| ABILITY TO FUNCTION AT WORK (Paid and/or volunteer) | No impairment | Mild impairment; some problems but generally able to function effectively | Moderate impairment | Severe impairment due to PTSD Sx, but able to minimally function | Completely unable to function occupationally because of PTSD |
|  | 5 | 4 | 3 | 2 | 1 |
| **WORK AND PRODUCTIVITY TOTAL** |  |  |  |  |  |

TEST DESCRIPTION AND SCORING

1) This is an observer rated instrument to be used only be mental health professionals trained and experienced in the clinical care of patients with PTSD. It was designed for use with combat veterans, particularly males.

2) This instrument is designed to compare functioning from the perspective of the patient and that of a co-habiting significant other (spouse/partner, parent, adult child,). Thus, the rater first interviews the patient and rates functioning, and then interviews the significant other and completes a separate rating. After these individual ratings are completed, the rater can discuss the findings with the two interviewees (for therapeutic purposes).

3) The wording of individual items are intended as guidelines. Raters should use their clinical judgment to estimate the level of functioning/impairment of function, taking into consideration the individual’s age, life circumstances, phase of life, and specific interests and opportunities.

4) The rater should rate functioning specifically in relationship to PTSD, considering the intrusive, avoidance/numbing and hyperarousal clusters to arrive at a single best functioning rating on each item***.*** Leaving a category blank is not permitted. If a given category does not apply (e.g., no children or grandchildren in the family to interact with; physical or financial limitations, etc.), a score of 3 should be given.

5) With respect to Section F, occupational functioning, there is less operational specification because of the variability in this category according to stage of life, preferences and other individual differences (e.g., people whose occupation is student/homemaking/childcare/domestic). The recommended method of scoring this category is for the examiner to access the patient’s current desired engagement in paid or unpaid productive activities and the influence of PTSD-related Sx on the ability to perform relative to the expected ability. Persons who are retired and currently engaging in only leisure activities can be asked about the influence of PTSD Sx on their occupational functioning in the past while working, and rated based on how their functioning at work might be affected given their current level of PTSD, but giving a rating of 3 in this case is also acceptable.

6). The past month should be considered the standard time period. In order to derive this optimally, a description of how patients’ functioning has changed from the past (pre-trauma or pretreatment) compared to currently can be obtained.

7) SCORING METHOD: Ratings are 1-5 for each of the 13 total items: A1, A2, A3, B 1, B2, B3, C 1, C2, C3, D 1, D2, E 1, F 1. Thus, scores can range for each category from 3-15 for A, 3-15 for B, 3-15 FOR C, 2-10 FOR D, 1-5 FOR E, 1-5 FOR F, AND 13-65 TOTAL.

| ***SCORING GUIDELINE IN CASES WHERE NO OPERATIONAL REFERENCE POINT FITS AND THE RATER’S GENREAL IMPRESSION MUST SUFFICE*** | | | | | |
| --- | --- | --- | --- | --- | --- |
|  | **EXCELLENT** | **GOOD** | **FAIR** | **NOT GOOD** | **POOR** |
| **Any item** | **5** | **4** | **3** | **2** | **1** |

The LFIPS was developed by Ralph J. Koek, MD, Staff Psychiatrist, Sepulveda Ambulatory Care Center, VA Greater Los Angeles Healthcare System and Clinical Professor, Department of Psychiatry and Biobehavioral Sciences, David Geffen School of Medicine at UCLA Los Angeles.

The LFIPS is available to mental health professionals for clinical use, or for research purposes

Contact:

Ralph J. Koek, MD

16111 Plummer St. (116A-11)

North Hills, CA 91343

800-516-4567, x7547

[ralph.koek@va.gov](mailto:ralph.koek@va.gov); [rkoek@ucla.edu](mailto:rkoek@ucla.edu)

***LIFE FUNCTIONING IN PTSD (LFIPS v2.0) SCORING SHEET***

| ITEM | Domain | Score |
| --- | --- | --- |
| A1 |  |  |
| A2 |  |  |
| A3 |  |  |
| A | FAMILY TOTAL |  |
| B1 |  |  |
| B2 |  |  |
| B3 |  |  |
| B | MARRIAGE TOTAL |  |
| C1 |  |  |
| C2 |  |  |
| C3 |  |  |
| C | CHILDREN TOTAL |  |
| D1 |  |  |
| D2 |  |  |
| D | FRIENDSHIPS TOTAL |  |
| E1 | LEISURE TOTAL |  |
| F1 | WORK AND PRODUCTIVITY TOTAL |  |
| **A-F** | **TOTAL SCORE** |  |

Score based on interview of Patient _____Family Member______Family Member name/Relationship _________________________

Patient Name/Study # _________________________________________Date______________Rater _________________________
