# Supplementary material for: Deep brain stimulation of the basolateral amygdala for treatment-refractory combat post-traumatic stress disorder (PTSD): study protocol for a pilot randomized controlled trial with blinded, staggered onset of stimulation
Source: Trials. 2014 Sep 10;15:356. doi: 10.1186/1745-6215-15-356 (PMC4168122; doi:10.1186/1745-6215-15-356)
Supplement: Supplementary file 3 — Additional file 3: Amygdala DBS in PTSD Scale (ADIPS): Scale developed for this study that consists of three standard measures for assessing Amygdala-related psychological functions and one inventory developed for this study to monitor behavioral, emotional, personality, sensory, perceptual and neurovegetative changes with amygdala DBS[168–170]. (DOCX 40 KB) [file 13063_2014_2226_MOESM3_ESM.docx]

This measure consists of 3 standardized neuropsychological assessments and a checklist of behavioral changes designed to assess specific amygdala related functions. Included are: The Faux Pas test [158]; International Affective Picture System from Center for the Study of Emotion and Attention (CSA) [159] at National Institutes of Mental Health (NIMH); Pictures of Facial Affect [160]; and amygdala related behavioral changes checklist.

1. FAUX PAS TEST

This clinician rated test was designed to assess theory of mind functioning. It consists of 20 scenarios in which something awkward or socially unacceptable is presented. The patient is then asked a series of questions to assess if he can properly identify the “faux pas” and understand why this is socially unacceptable. Points are given for correct answers, 60 being the highest score and 0 the lowest.

2. INTERNATIONAL AFFECTIVE PICTURE SYSTEM

This is a database of pictures used to elicit and monitor a range of different emotions. It was developed by the [National Institute of Mental Health](http://en.wikipedia.org/wiki/National_Institute_of_Mental_Health) Center for Emotion and Attention at the [University of Florida](http://en.wikipedia.org/wiki/University_of_Florida). It is used to assess emotional responses to visual stimuli, a function which is mediated by the Amygdala.

3. PICTURES OF FACIAL AFFECT

This test was designed to assess the ability to recognize facial expression related to 6 cardinal emotions - happiness, sadness, anger, fear, disgust and surprise. It consists of 110 standard photographs of facial expressions which the patient is presented with and has 10 seconds to answer which emotion the facial expression corresponds with.

4. AMYGDALA RELATED BEHAVIORAL CHANGES INVENTORY

This clinician administered monitoring parameter was developed for this study to assess behavioral, emotional, personality, sensory, perceptual and neurovegetative changes before and after deep brain stimulation. Items were compiled from an extensive literature review of functions of the Amygdala, and possible change with lesion or stimulation of the Amygdala. The scale assesses both presence of a change or side effect and severity of this change

***Amygdala Related Behavioral Changes (ARBC) Inventory***

|  | BEHAVIORS OBSERVED BY PATIENT, SIGNIFICANT OTHERS OR CLINICIANS IN THE PAST MONTH | Sev | | Mod | | | | | | Mild | NONE/  BASE-LINE | Mild | Mod | | Sev | |
| --- | --- | --- | --- | --- | --- | --- | --- | --- | --- | --- | --- | --- | --- | --- | --- | --- |
|  |  | LESS | | | | | | | | |  | MORE | | | | |
| **Item #** | | ***A. CLASSIC PERSONALITY CHANGES ASSOCIATED WITH TEMPORAL LOBE DAMAGE*** | | | | | | | | | | | | | | |
| **1** | HYPO/HYPERRELIGIOSITY | -3 | -2 | | | | | | -1 | | 0 | 1 | | 2 | | 3 |
| **2** | INCREASED PHILOSPHICAL CONCERN | -3 | -2 | | | | | | -1 | | 0 | 1 | | 2 | | 3 |
| **3** | HYPO/HYPERGRAPHIA | -3 | -2 | | | | | | -1 | | 0 | 1 | | 2 | | 3 |
| **4** | HYPO/HYPERMORALISM | -3 | -2 | | | | | | -1 | | 0 | 1 | | 2 | | 3 |
| **5** | HYPO/HYPEREMOTIONALITY | -3 | -2 | | | | | | -1 | | 0 | 1 | | 2 | | 3 |
| **6** | HYPO/HYPERSEXUALITY | -3 | -2 | | | | | | -1 | | 0 | 1 | | 2 | | 3 |
| **7** | INTERPERSONAL “STICKINESS” | -3 | -2 | | | | | | -1 | | 0 | 1 | | 2 | | 3 |
| **8** | CIRCUMSTANTIALITY | -3 | -2 | | | | | | -1 | | 0 | 1 | | 2 | | 3 |
| **9** | HYPERORALITY | -3 | -2 | | | | | | -1 | | 0 | 1 | | 2 | | 3 |
| **10** | HYPERMETAMORPHOSIS | -3 | -2 | | | | | | -1 | | 0 | 1 | | 2 | | 3 |
|  | ***Personality Subtotal*** | -30 | -20 | | | | | | -10 | | 0 | 10 | | 20 | | 30 |
|  | | ***B. EMOTIONAL EXPERIENCES UNRELATED TO ENVIRONMENTAL CUES*** | | | | | | | | | | | | | | |
| **1** | *Fear/Panic* | -6 | -4 | | | | | | -2 | | 0 | 2 | | 4 | | 6 |
| **2** | *Dread* | -3 | -2 | | | | | | -1 | | 0 | 1 | | 2 | | 3 |
| **3** | *Doom* | -3 | -2 | | | | | | -1 | | 0 | 1 | | 2 | | 3 |
| **4** | *Elation* | -3 | -2 | | | | | | -1 | | 0 | 1 | | 2 | | 3 |
| **5** | *Sadness* | -3 | -2 | | | | | | -1 | | 0 | 1 | | 2 | | 3 |
| **6** | *Sexual arousal/orgasm* | -3 | -2 | | | | | | -1 | | 0 | 1 | | 2 | | 3 |
| **7** | *Someone’s Presence* | -3 | -2 | | | | | | -1 | | 0 | 1 | | 2 | | 3 |
| **8** | *Serenity/Calmness* | -3 | -2 | | | | | | -1 | | 0 | 1 | | 2 | | 3 |
| **9** | *Other (Specify___________________* | -3 | -2 | | | | | | -1 | | 0 | 1 | | 2 | | 3 |
|  | ***Emotion Subtotal*** | -30 | -20 | | | | | | -10 | | 0 | 10 | | 20 | | 30 |
|  | | ***C. SENSORY CHANGES*** | | | | | | | | | | | | | | |
| **1** | OLFACTION | -3 | -2 | | | | | | -1 | | 0 | 1 | | 2 | | 3 |
| **2** | TASTE | -3 | -2 | | | | | | -1 | | 0 | 1 | | 2 | | 3 |
| **3** | TOUCH | -3 | -2 | | | | | | -1 | | 0 | 1 | | 2 | | 3 |
| **4** | HEARING | -3 | -2 | | | | | | -1 | | 0 | 1 | | 2 | | 3 |
| **5** | BALANCE | -3 | -2 | | | | | | -1 | | 0 | 1 | | 2 | | 3 |
| **6** | VISION | -3 | -2 | | | | | | -1 | | 0 | 1 | | 2 | | 3 |
|  | ***Sensory Subtotal*** | -18 | -12 | | | | | | -6 | | 0 | 6 | | 12 | | 18 |
|  | | ***D. PERCEPTUAL ALTERATIONS*** | | | | | | | | | | | | | | |
| **1** | OUT OF BODY EXPERIENCE | -3 | -2 | | | | | | -1 | | 0 | 1 | | 2 | | 3 |
| **2** | HYPERMETAMORPHOSIS | -3 | -2 | | | | | | -1 | | 0 | 1 | | 2 | | 3 |
| **3** | MICROPSIA | -3 | -2 | | | | | | -1 | | 0 | 1 | | 2 | | 3 |
| **4** | MACROPSIA | -3 | -2 | | | | | | -1 | | 0 | 1 | | 2 | | 3 |
| **5** | METAMORPHOPSIA | -3 | -2 | | | | | | -1 | | 0 | 1 | | 2 | | 3 |
| **6** | VISUAL OBJECT AGNOSIA | -3 | -2 | | | | | | -1 | | 0 | 1 | | 2 | | 3 |
| **7** | DÉJÀ VU/JAMAIS VU/DÉJÀ ENTENDU/JAMAIS ENTENDU/DÉJÀ PENSEE | -3 | -2 | | | | | | -1 | | 0 | 1 | | 2 | | 3 |
| **8** | FUGUE | -3 | -2 | | | | | | -1 | | 0 | 1 | | 2 | | 3 |
| **9** | HALLUCINATIONS: *Visual* | -6 | -4 | | | | | | -2 | | 0 | 2 | | 4 | | 6 |
| **10** | HALLUCINATIONS: *Auditory* | -6 | -4 | | | | | | -2 | | 0 | 2 | | 4 | | 6 |
| **11** | HALLUCINATIONS: *Somatosensory* | -6 | -4 | | | | | | -2 | | 0 | 2 | | 4 | | 6 |
| **12** | HALLUCINATIONS: *Olfactory* | -6 | -4 | | | | | | -2 | | 0 | 2 | | 4 | | 6 |
| **13** | HALLUCINATIONS: *Gustatory* | -6 | -4 | | | | | | -2 | | 0 | 2 | | 4 | | 6 |
| **14** | DELUSIONS | -6 | -4 | | | | | | -2 | | 0 | 2 | | 4 | | 6 |
|  | ***Perceptual Subtotal*** | -60 | -40 | | | | | | -20 | | 0 | 20 | | 40 | | 60 |
|  | | ***E. SPECIFIC BEHAVIORAL CHANGES*** | | | | | | | | | | | | | | |
| **1** | FORCED THINKING | -3 | | | -2 | | | -1 | | | 0 | 1 | | 2 | | 3 |
| **2** | FORCED SPEAKING | -3 | | | -2 | | | -1 | | | 0 | 1 | | 2 | | 3 |
| **3** | FORCED RUNNING | -3 | | | -2 | | | -1 | | | 0 | 1 | | 2 | | 3 |
| **4** | FORCED WANDERING (PORIOMANIA) | -3 | | | -2 | | | -1 | | | 0 | 1 | | 2 | | 3 |
| **5** | SPEECH ARREST | -3 | | | -2 | | | -1 | | | 0 | 1 | | 2 | | 3 |
| **6** | MOTOR AUTOMATISMS (Specify ____________________________ | -3 | | | -2 | | | -1 | | | 0 | 1 | | 2 | | 3 |
| **7** | PARAPHILIAS | -6 | | | -4 | | | -2 | | | 0 | 2 | | 4 | | 6 |
| **8** | VERBAL AGGRESSIVENESS | -6 | | | -4 | | | -2 | | | 0 | 2 | | 4 | | 6 |
| **9** | PHYSICAL AGGRESSIVENESS TOWARD INANIMATE OBJECTS | -12 | | | -8 | | | -4 | | | 0 | 4 | | 8 | | 12 |
| **10** | PHYSICAL AGGRESSIVENESS TOWARD ANIMALS | -12 | | | -8 | | | -4 | | | 0 | 4 | | 8 | | 12 |
| **11** | PHYSICAL AGGRESSIVENESS TOWARD SELF (NON-SUICIDAL) | -24 | | | -16 | | | -8 | | | 0 | 8 | | 16 | | 24 |
| **12** | PHYSICAL AGGRESSIVENESS TOWARD OTHERS | -24 | | | -16 | | | -8 | | | 0 | 8 | | 16 | | 24 |
| **13** | OTHER NEW BEHVIORS  Specify_________________ | -6 | | | -4 | | | -2 | | | 0 | 2 | | 4 | | 6 |
|  | ***Behavior Subtotal*** | -108 | | | -72 | | | -36 | | |  | 36 | | 72 | | 108 |
|  | | ***F. VEGETATIVE FUNCTIONS*** | | | | | | | | | | | | | | |
| **1** | APPETITE | -6 | -4 | | | -2 | | | | | 0 | 2 | | 4 | | 6 |
| **2** | SLEEP | -6 | -4 | | | -2 | | | | | 0 | 2 | | 4 | | 6 |
|  | ***Vegetative Functions Subtotal*** | -12 | -8 | | | -4 | | | | | 0 | 4 | | 8 | | 12 |
|  |  | ***POSSIBLE TOTALS FOR EACH CATEGORY*** | | | | | | | | | | | | | | |
| **A** | ***Personality*** | -30 | | | -20 | | -10 | | | | 0 | 10 | | 20 | | 30 |
| **B** | ***Emotional*** | -30 | | | -20 | | -10 | | | | 0 | 10 | | 20 | | 30 |
| **C** | ***Sensory*** | -18 | | | -12 | | -6 | | | | 0 | 6 | | 12 | | 18 |
| **D** | ***Perceptual*** | -60 | | | -40 | | -20 | | | | 0 | 20 | | 40 | | 60 |
| **E** | ***Behavioral*** | -108 | | | -72 | | -36 | | | | 0 | 36 | | 72 | | 108 |
| **F** | ***Vegetative*** | -12 | | | -8 | | -4 | | | | 0 | 4 | | 8 | | 12 |
|  |  |  | | |  | |  | | | |  |  | |  | |  |
|  | ***Total Amygdala-Related Behavioral Changes (ARBC) Score*** | -258 | | | -172 | | -86 | | | | 0 | 86 | | 172 | | 258 |
|  |  |  | | |  | |  | | | |  |  | |  | |  |

**Test administration, scoring and advisement**

1) This is a semi-structured, observer-rated instrument that should be administered by a clinical neuroscientist familiar with the nature of the functions in each category.

2) The battery should be administered by interviewing the patient in the presence of a significant other preferably cohabiting with the patient. The rating clinician should make sure the patient and informant understand the nature of the phenomena being inquired about (See ***ARBC Glossary*** for definition of terms).

3) Ratings should correspond to ***the past month or other specified time interval only.***

4) There is no total ADIPS Score. Each of the four subtests are scored separately according to published guidelines that accompany the standard tests (Items 1-3) or for Item 4, the ARBC, as above. The ARBC Scoring Record can be used to record ARBC ratings at each assessment.

5) The ***ARBC Scoring Record*** permits recording of the total scores in each category, as well as the specific items that have changed, and items that have become newly emergent behaviors since the last visit. On the scoring sheet, items rated less, more or new can be specifically recorded (e.g., A1, A4, …) and the total # of items in each change category can also be recorderd. This permits both quantitative and qualitative comparisons between ratings.

6) Operationalized rating guidelines for severity differ according to the specific items as shown in the ***ARBC Rating Guideline*** below.

7) The ARBC was created in 2010 and modified in 2013 by Ralph J. Koek, MD, Staff Psychiatrist at the VA Greater Los Angeles Healthcare System and Clinical Professor, David Geffen School of Medicine at UCLA. It can be used freely by health care professionals for clinical or educational purposes. For further information, contact Dr. Koek: [ralph.koek@va.gov](mailto:ralph.koek@va.gov) or [rkoek@ucla.edu](mailto:rkoek@ucla.edu) or Tel 800-516-4567, x7547

REFERENCES:

158. Stone VE, Baron-Cohen S, Calder A, Keane J, Young A. Acquired theory of mind

impairments in individuals with bilateral amygdala lesions. Neuropsychologia

2003;41:209-220.

159. Lang, P.J., Bradley, M.M., & Cuthbert, B.N. (2008). International affective picture system

(IAPS): Affective ratings of pictures and instruction manual. Technical Report A-8. University of Florida, Gainesville, FL.160. Ekman P: **Pictures of Facial Affect (POFA)** [http://www.paulekman.com/product/pictures-of-facial-affect-pofa/]

***ARBC Rating Guideline***

**For Items A 1-8, C 1-6, F 1-2^a^**

| Rating | Operationalized Severity/Intensity Descriptor |
| --- | --- |
| -3 | substantially and definitely worse than baseline, with resulting improvement in important life function(s) |
| -2 | substantially and definitely worse than baseline, but No effect on function |
| -1 | Mildly or possibly worse than baseline, but not at all bothersome |
| 0 | No change from baseline |
| 1 | Mildly or possibly better than baseline, but No effect on function |
| 2 | substantially and definitly better than baseline, but No effect on function |
| 3 | substantially and definitly better than baseline, with resulting improvement in important life function(s) |

a. For items F 1 and 2, substitute the word less for worse, and more for better.

**For items A 9-10, B 1-9,D 1-14, E 1-13^b^**

| Rating | Operationalized Severity/Intensity Descriptor |
| --- | --- |
| -3 | One or more occasions with definite distress or impairment |
| -2 | More than one occasion without functional impairment or distress |
| -1 | One Occasion |
| 0 | None |
| 1 | Possibly or barely less frequent than before |
| 2 | Definitely less frequent than before, but no significant change in functioning or subjective distress |
| 3 | Less frequent than before and associated with improvement in functioning or subjective distress. |

b. Note that for some items rated using this guideline (eg D7, E7-12) there could be an ongoing frequency of the behavior at baseline. Thus, the 1,2 and 3 ratings apply, but the -3, -2 and -1 do not. For these items, substitute the following:

| -3 | Definite increase in frequency or intensity with definite distress or impairment |
| --- | --- |
| -2 | Definite increase in frequency or intensity without functional impairment or distress |
| -1 | Mild or possibly increased frequency or intensity |

***ARBC Glossary***

| **Item #** | **Phenomenon** | **Definition** |
| --- | --- | --- |
| A1 | Hypo/hyperreligiosity | Degree to which religious/theological beliefs or actions are important to the person’s life and world view, based on statements, activities, writings and subjective experience. |
| A2 | Increased Philsophical Concern | Tendency to think/talk about the meaning, source, definition, logic etc of experiences, ideas, events or concepts |
| A3 | Hypo/hypergraphia | Tendency to write things down, especially thoughts, concerns or ideas, for self or for communication to others. Rate mainly the quantity of output. |
| A4 | Hypo/hypermoralism | Tendency to express concern about the moral principles (Right and wrong) involved in interpersonal experiences, and there meaning in terms of justice, higher being, etc. |
| A5 | Hypo/hyperemotionality | Intensity of expressed/exhibited/experienced emotional reaction relative to the event triggering the emotion. Much include degree of actual change in feelings. Do not rate just verbal descriptions of feelings. |
| A6 | Hypo/hypersexuality | Expressed interest or tendency to engage in sexual activity OR inclusion of sexual matters in content of speech |
| A7 | Interpersonal “Stickiness” | Tendency to persist in a conversation or stay in the presence of others—friends, strangers, physician’s office—after subtle or overt communication has been made that it is time for the interaction to end. |
| A8 | Circumstantiality | Tendency to include excess detail when describing events, answering questions, etc. |
| A9 | Hyperorality | Placing non-food objects in mouth (other than culturally sanctioned items such as a pen, glasses, fingernails). |
| A10 | Hypermetamorphosis | Forced attention to environmental stimuli |
| B1 | Fear/Panic | Emotional ***experiences*** unrelated to environmental cues. Usually sudden and brief. For these items, examiner should start out with an open-ended question such as “in the past month, have you suddenly feeling different inside for no apparent reason? If yes, then get detailed description from the patient, and see if the experience matches any of the listed categories or B9. Then, examiner should ask the patient whether in the past month he has had any of these experiences and rate accordingly. |
| B2 | Dread |  |
| B3 | Doom |  |
| B4 | Elation |  |
| B5 | Sadness |  |
| B6 | Sexual arousal/orgasm |  |
| B7 | Someone’s Presence |  |
| B8 | Serenity/calmness |  |
| B9 | Other |  |
| C1 | Olfaction | In this section, rate the individual’s subjective experience in the effectiveness (quantity) or quality of their sensory functioning in each modality. These items refer to HOW the patient experiences a sensation, rather than whether they have had any particular experience, as in items D9-13. |
| C2 | Taste |  |
| C3 | Touch |  |
| C4 | Hearing |  |
| C5 | Balance |  |
| C6 | Vision |  |
| D1 | Out of Body | Experience of being detached from one’s surroundings, being “on the outside looking in,” etc. |
| D2 | Synesthesia | sensation that normally occurs in one sense modality occurs when another modality is stimulated: hearing red, smelling music, etc. |
| D3 | Micropsia | Experience that objects in visual world are smaller than previously or than expected |
| D4 | Macropsia | Experience that objects in visual world are larger than previously or larger than expected |
| D5 | Metamorphopsia | Change in the qualities of an actually perceived visual object: size, shape, position, motion, number, or relationship to the observer (wavy edges, apparent motion of a tree, narrowing or widening, etc. Experiences D2,D3,D4, D7, OR D9-13 should not be rated here. |
| D6 | Visual Object Agnosia | Inability to *understand*  the nature of a specific object, even if it can be named. Do not rate anomia or word finding difficulty here. |
| D7 | Déjà vu | Experience that one is seeing something familiar, when it should be unfamiliar |
| D7 | Jamais vu | Sense that one is seeing something unfamiliar, when it should be familiar |
| D7 | Deja Entendu | Experience that one is hearing something familiar, when it should be unfamiliar |
| D7 | Jamais Entendu | Sense that one is hearing something unfamiliar, when it should be familiar |
| D7 | Deja Pensee | Sense that one is thinking something familiar, when it should be unfamiliar |
| D7 | Jamais Pensee | Experience that one is thinking something unfamiliar, when it should be familiar |
| D8 | Fugue | Describes phenomenon of being awake and physically functional, while mentally operating as if in a state other than the usual conscious experience. May include leaving normal surroundings, getting lost and being picked up by authorities in extreme cases. A probe question is “have you had the experience of losing time?” with further questioning required to understand the nature of the event if answered affirmatively. |
| D9 | Visual Hallucinations | For this question most patients will be able to answer accurately if asked “Have you had any hallucinations?” This is preferable to asking “Have you seen things that were not there?” Visual Hall must be distinguished from mental images, thoughts or beliefs, and even sometimes from aud hall because the patient may believe there MUST have been someone present when they heard a voice. |
| D10 | Auditory Hallucinations | The best probe question is “have you heard things that others wo were present told you they did not hear?” Also, “have you had the experience of being alone somewhere and then hearing a person’s voice speak to you out loud?” “Do you hear voices?” is not the recommended primary probe. |
| D11 | Somatosensory Hallucinations | Experience of someone or something touching one’s body when no corresponding person or object can be seen. If the only such somatosensory experience is sexual in nature, rate under item B6 instead of here. |
| D12 | Olfactory Hallucinations | Smells for which no source could be discerned, and/or which others who were present could not detect. |
| D13 | Gustatory Hallucinations | but spontaneous sensation of tasting something when nothing was put in the mouth (not change in the taste of food items which should be rated in C2) |
| E1 | Forced Thinking | Self-explanatory |
| E2 | Forced Speaking | Self-explanatory |
| E3 | Forced Running | Self-explanatory |
| E4 | Forced Wandering (Poriomania) | Self-explanatory |
| E5 | Speech Arrest | Self-explanatory |
| E6 | Motor automatisms | Repetitive, purposeless movements |
| E7 | Paraphilias |  |
| E8 | Verbal Aggressiveness | In order to rate these items, interviewer has to use a combination of open-ended and direct questions, obtaining descriptions of any actual events. |
| E9 | Physical Aggression toward Inanimate Objects |  |
| E10 | Physical Aggression toward animals |  |
| E11 | Physical Aggression toward self (non-suicidal) |  |
| E12 | Physical Aggression toward other people |  |
| F1 | Appetite | Rate only the general intensity of appetite and amount of food eaten. Do not rate changes in food preference here. Make note if the patient has made a conscious decision to change his weight or diet for specific reasons |
| F2 | Sleep | Rate based on the AMOUNT not the subjective quality, or other aspects like nightmares, difficulty falling asleep, difficulty staying asleep, etc.. |

***Amygdala Related Behavioral Changes (ARBC) Inventory Scoring Record***

| Patient Name/ID #: _______________ | Date: ____________ | Rater: _____________________ |
| --- | --- | --- |
|  | | |

| Category | Total | Items Rated Less (#) | Items Rated More (#) | New Items (#) |
| --- | --- | --- | --- | --- |
| **A *Personality*** |  |  |  |  |
| **B *Emotion*** |  |  |  |  |
| **C *Sensation*** |  |  |  |  |
| **D *Perception*** |  |  |  |  |
| **E *Behavior*** |  |  |  |  |
| **F *VegetativeFunction*** |  |  |  |  |
|  | | | | |
| ***Total ARBC Score*** |  |  |  |  |
|  |  |  |  |  |
