# Supplementary material for: Deep brain stimulation of the basolateral amygdala for treatment-refractory combat post-traumatic stress disorder (PTSD): study protocol for a pilot randomized controlled trial with blinded, staggered onset of stimulation
Source: Trials. 2014 Sep 10;15:356. doi: 10.1186/1745-6215-15-356 (PMC4168122; doi:10.1186/1745-6215-15-356)
Supplement: Supplementary file 4 — Additional file 4: Neuropsychological Battery: List of standardized assessments of attention, concentration, language, memory, visuospatial and executive function to be administered at intervals during study[171–184]. (DOCX 16 KB) [file 13063_2014_2226_MOESM4_ESM.docx]

**Koek et al. Amygdala DBS in PTSD. Additional File 4. Neuropsychological Assessment Battery**

ATTENTION, CONCENTRATION & MEMORY

Wechsler Adult Intelligence Scale IV Digit Span & Digit Symbol [169]

Controlled Oral Word Association Test [170]

Trail Making Tests A and B [171]

Rey-15 Test of Mental Effort [172]

Ruff Figural Fluency Test [173]

Rey Auditory Verbal Learning Test [174]

California Verbal Learning Test [175]

LANGUAGE

Wechsler Test of Adult Reading [169]

VISUOSPATIAL FUNCTION

Hooper Visual Organization Test [176]

Rey-Osterrieth Complex Figure [177]

Clock Drawing [178]

FRONTAL SYSTEMS/EXECUTIVE FUNCTIONS

Wisconsin Card Sorting Test [179]

Stroop Color and Word test [180]

Iowa Gambling Task [181]

GENERAL

Mini Mental State Exam [182]
