# Supplementary material for: Deep brain stimulation of the basolateral amygdala for treatment-refractory combat post-traumatic stress disorder (PTSD): study protocol for a pilot randomized controlled trial with blinded, staggered onset of stimulation
Source: Trials. 2014 Sep 10;15:356. doi: 10.1186/1745-6215-15-356 (PMC4168122; doi:10.1186/1745-6215-15-356)
Supplement: Supplementary file 5 — Additional file 5: Study Timeline.(DOCX 41 KB) [file 13063_2014_2226_MOESM5_ESM.docx]

**CLINICIAN REFERRAL** **SELF-REFERRAL**

NO

CHART REVIEW-BASED I/E CRITERIA? EXCLUDE

YES

NO

ADDITIONAL I/E CRITERIA MET EXCLUDE

YES

**CONSENT**

BASELINE NEUROPSYCH BATTERY; PET/CT

SURGERY + 3 DAY HOSPITALIZATION

Stimulators OFF x 1 month

EEG TELEMETRY SESSION

RANDOMIZATION

**STIMULATORS ON (N =3)** **SHAM STIMULATION (N=3) X 2 MONTHS**

THEN STIMULATORS ON

WEEKLY CLINICAL ASSESSMENTS X 5 MONTHS

MONTHLY STUDY OUTCOME MEASURES AND STIMULATOR ADJUSTMENTS X 15 MONTHS THEN EVERY 3 MONTHS X 9 MONTHS

REPEAT NEUOPSYCHOLOGICAL BATTERY AT 6, 12 AND 24 MONTHS

REPEAT PET/CT AT 12-15 MONTHS

STUDY END; CLINICAL FOLOW-UP, BATTERY REPLACEMENT
