# Supplementary figures and images for: Deep brain stimulation of the basolateral amygdala for treatment-refractory combat post-traumatic stress disorder (PTSD): study protocol for a pilot randomized controlled trial with blinded, staggered onset of stimulation
Source: Trials. 2014 Sep 10;15:356. doi: 10.1186/1745-6215-15-356 (PMC4168122; doi:10.1186/1745-6215-15-356)

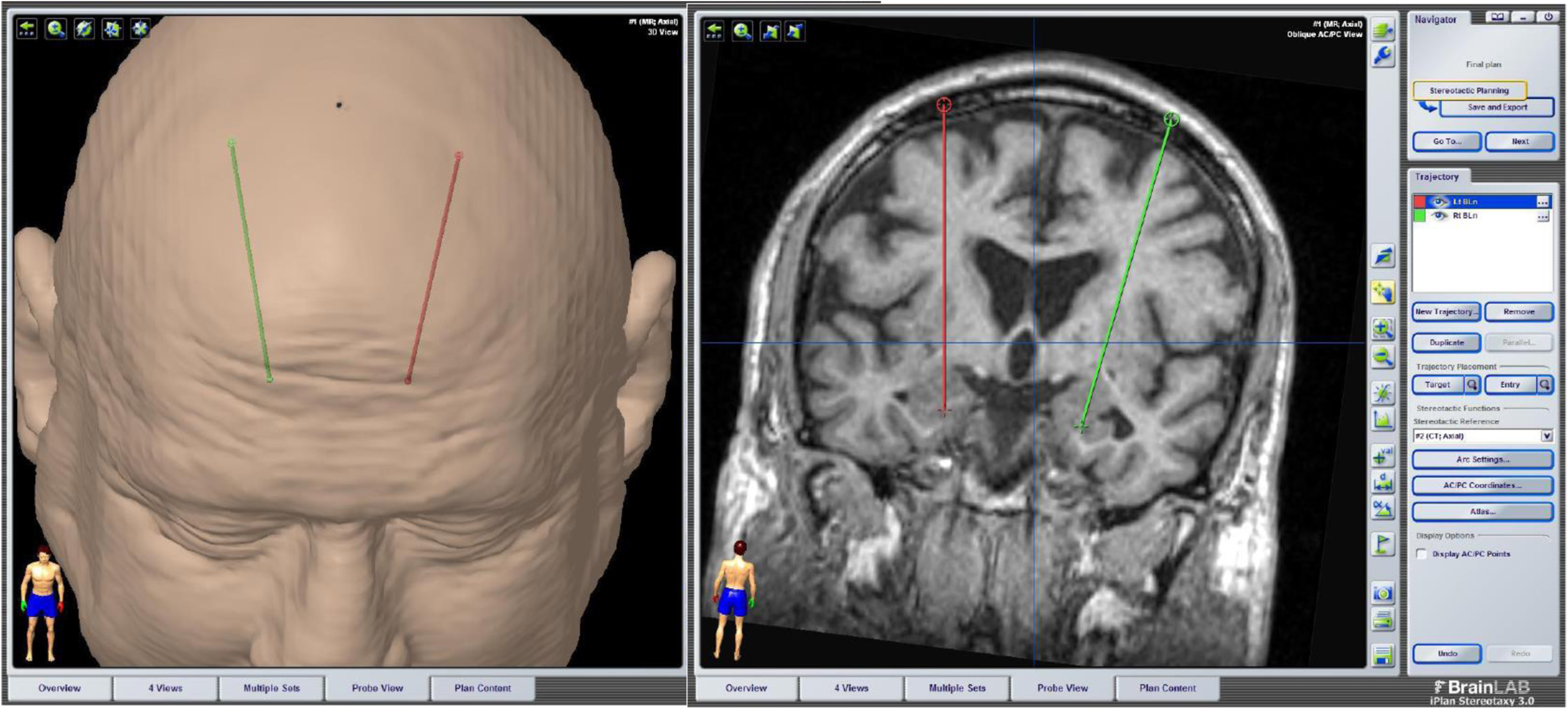

Supplement: Supplementary file 6 — Authors’ original file for figure 1 [file 13063_2014_2226_MOESM6_ESM.tif]
